# The genome and transcriptome of *Haemonchus contortus*, a key model parasite for drug and vaccine discovery

**DOI:** 10.1186/gb-2013-14-8-r88

**Published:** 2013-08-28

**Authors:** Roz Laing, Taisei Kikuchi, Axel Martinelli, Isheng J Tsai, Robin N Beech, Elizabeth Redman, Nancy Holroyd, David J Bartley, Helen Beasley, Collette Britton, David Curran, Eileen Devaney, Aude Gilabert, Martin Hunt, Frank Jackson, Stephanie L Johnston, Ivan Kryukov, Keyu Li, Alison A Morrison, Adam J Reid, Neil Sargison, Gary I Saunders, James D Wasmuth, Adrian Wolstenholme, Matthew Berriman, John S Gilleard, James A Cotton

**Affiliations:** 1 Institute of Infection, Immunity and Inflammation, College of Medical, Veterinary and Life Sciences, University of Glasgow, 464 Bearsden Road, Glasgow, Scotland, G61 1QH, UK; 2 Wellcome Trust Sanger Institute, Wellcome Trust Genome Campus, Hinxton, Cambridge, CB10 1SA, UK; 3 Division of Parasitology, Department of Infectious Disease, Faculty of Medicine, University of Miyazaki, Miyazaki, 889-1692 Japan; 4 Institute of Parasitology, Macdonald Campus, McGill University, 21,111 Lakeshore Road, Ste. Anne de Bellevue, Québec, Canada H9X 3V9; 5 Department of Comparative Biology and Experimental Medicine, Faculty of Veterinary Medicine, Faculty of Veterinary Medicine, 3330 Hospital Drive NW, Calgary, Alberta, Canada T2N 1N4; 6 Disease Control, Moredun Research Institute, Pentlands Science Park, Bush Loan, Penicuik, Midlothian, EH26 0PZ, UK; 7 Department of Ecosystem and Public Health, Faculty of Veterinary Medicine, Faculty of Veterinary Medicine, 3330 Hospital Drive NW, Calgary, Alberta, Canada T2N 1N4; 8 Royal (Dick) School of Veterinary Studies, University of Edinburgh, Easter Bush Veterinary Centre, Roslin, Midlothian EH25 9RG, Scotland, UK; 9 Department of Infectious Diseases and Center for Tropical and Emerging Global Disease, University of Georgia, Athens, Georgia 30602, USA

## Abstract

**Background:**

The small ruminant parasite *Haemonchus contortus *is the most widely used parasitic nematode in drug discovery, vaccine development and anthelmintic resistance research. Its remarkable propensity to develop resistance threatens the viability of the sheep industry in many regions of the world and provides a cautionary example of the effect of mass drug administration to control parasitic nematodes. Its phylogenetic position makes it particularly well placed for comparison with the free-living nematode *Caenorhabditis elegans *and the most economically important parasites of livestock and humans.

**Results:**

Here we report the detailed analysis of a draft genome assembly and extensive transcriptomic dataset for *H. contortus*. This represents the first genome to be published for a strongylid nematode and the most extensive transcriptomic dataset for any parasitic nematode reported to date. We show a general pattern of conservation of genome structure and gene content between *H. contortus *and *C. elegans*, but also a dramatic expansion of important parasite gene families. We identify genes involved in parasite-specific pathways such as blood feeding, neurological function, and drug metabolism. In particular, we describe complete gene repertoires for known drug target families, providing the most comprehensive understanding yet of the action of several important anthelmintics. Also, we identify a set of genes enriched in the parasitic stages of the lifecycle and the parasite gut that provide a rich source of vaccine and drug target candidates.

**Conclusions:**

The *H. contortus *genome and transcriptome provide an essential platform for postgenomic research in this and other important strongylid parasites.

## Background

Resistance to broad spectrum anthelmintic drugs is now widespread in parasites of domestic livestock [1,2] and there are increasing concerns about the sustainability of human parasite control programs using mass administration of the same drugs [3]. Consequently, there is an urgent need to understand the genetic mechanisms underlying anthelmintic resistance and to discover new methods of chemical and non-chemical control. However, the genomic and genetic resources required to underpin research in parasitic nematodes are lacking [4-6]. The free-living nematode *Caenorhabditis elegans *is a powerful model system, but it has clear limitations for the study of parasitic species [7]. Although the need to develop workable parasitic nematode model systems is widely recognized, most human helminth species are not amenable to experimental study. In contrast, *Haemonchus contortus*, a gastrointestinal parasitic nematode of small ruminants, has a successful track record in anthelmintic resistance [7,8], drug discovery [9,10] and vaccine [11-13] research. It is amongst the most experimentally tractable parasites for a number of reasons: adult females are relatively large and produce thousands of eggs per day, allowing the production of large amounts of biological and genetic material, the infective larvae (L3) can be viably cryopreserved, and *in vivo *studies, including genetic crosses, can be undertaken in the natural host [8]. Its phylogenetic position within the most closely related group of parasites to *C. elegans *facilitates comparative genomics and heterologous gene expression, allowing functional studies to be performed on *H. contortus *genes and regulatory elements [4,14]. As this parasite is a strongylid nematode, research on it is of particular relevance to the most economically significant parasites of grazing ruminants and to the human hookworms [15]. *H. contortus *itself causes major economic loss in small ruminants worldwide; it is highly pathogenic and unsurpassed in its ability to develop resistance to every anthelmintic used in its control. Notably, these include a number of core drugs used for mass drug administration programs in humans [3]. Anthelmintic resistance in *H. contortus *and related strongylids now threatens the viability of the sheep industry throughout the world [2]. This represents both a warning and a useful model for the consequences of the widespread intensive use of anthelmintics that are now being used to control human parasites in the developing world [3].

*H. contortus *has a direct and rapid lifecycle (Figure S1 in Additional file 1); adults reside and mate in the abomasum of the ruminant host, then females produce eggs to be excreted in the feces. The eggs embryonate, develop and hatch as first stage larvae (L1), develop and molt to become second stage larvae (L2), then molt again to become third stage larvae (L3) in the feces. The L3 migrate onto pasture to be ingested by the grazing ruminant host. The L3 shed the retained L2 cuticle (exsheath) in the rumen, travel to the abomasum and develop through to the fourth larval stage (L4) then adults in two to three weeks [16]. As voracious blood-feeders, *H. contortus *L4 and adults can cause severe hemorrhagic gastritis, hypoproteinemia, anemia and edema, with acute infections resulting in death of the animal host. One adult female can produce up to 10,000 eggs per day [17] and a single animal can harbor thousands of worms. This extremely high fecundity, under conducive host and environmental conditions, can give rise to population explosions and devastating disease outbreaks. Development occurs most rapidly in warm humid conditions, but the L4 stage can undergo arrested development within the host to survive adverse conditions such as prolonged drought or cold winters [18]. This feature of facultative arrested development, aided by movement of domestic livestock and climate change, has ensured the worldwide distribution and success of this parasite even though its evolutionary origins were in sub-Saharan Africa [19].

One striking feature of *H. contortus*, in common with related nematode species, is the extremely high level of genetic diversity that has been reported in both laboratory and field populations; this is thought to be predominantly a function of its large effective population size [20-22]. Genetic variation may underlie both the parasite's remarkable ability to adapt to different climatic regions and host species [23] and its alarming propensity to develop drug resistance. This high level of genetic polymorphism has also provided a major challenge to genome assembly, necessitating the production of an inbred line from which to prepare DNA template.

In this paper, we describe the assembly and annotation of the draft genome of MHco3(ISE).N1, a genetically well characterized inbred *H. contortus *strain that is susceptible to all major anthelmintic drugs. The most extensive comparative transcriptomic sequencing and analysis yet described for a parasitic nematode was undertaken on representative life-stages and the nematode gut to explore different aspects of nematode biology, evolution and parasitism. This draft genome provides a platform for future research in anthelmintic resistance, drug discovery and vaccine development using *H. contortus*, currently the most important experimental model for the strongylid nematode group.

## Results and discussion

### Genome structure and content

We assembled a draft sequence of the *H. contortus *genome based on data from a mixture of sequencing technologies (Material and methods; Additional results and methods in Additional file 1). Our final draft assembly consists of 67,687 contigs linked into 26,044 scaffolds of total length 370 Mb, including 23.8 Mb of inferred gaps between contigs, with an average and N50 scaffold length of 14,206 and 83,287 bp, respectively (Table S1 in Additional file 1). This is a significantly larger genome size than the approximately 60 Mb predicted with flow cytometry [24] but it is consistent with a prediction of approximately 200 to 300 Mb inferred from a pilot annotation of two large contiguous regions of the genome [14], making this the largest nematode genome sequenced to date (Table S1 in Additional file 1). The overall base composition of all sequence contigs was close to neutral at a mean 41.3% GC, slightly higher than that for other clade V nematodes. Approximately 93% of conserved eukaryotic genes can be identified in the assembly, suggesting that our draft assembly represents at least that fraction of the *H. contortus *genome [25], as many of these models are incomplete or split between contigs (Table S2 in Additional file 1). Despite this, our current assembly is of similar quality to some other published draft nematode genome sequences [26-30] (Table S1 in Additional file 1).

**Figure 1:**
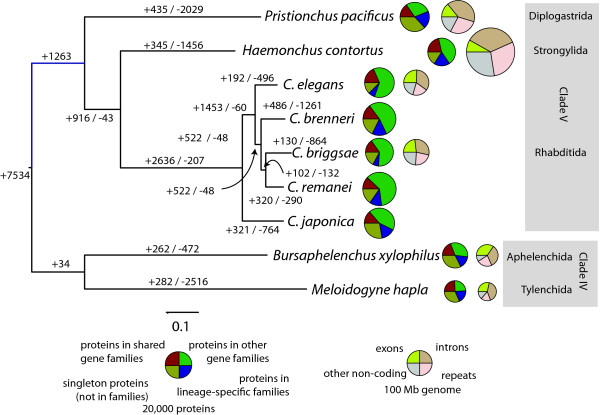
**Evolution of genome content in clade V nematodes**. A maximum-likelihood phylogeny based on concatenated alignment of single-copy genes. Values on edges represent the inferred numbers of births (+) and deaths (-) of gene families along that edge. Note that our approach cannot distinguish gene family losses from gains on the basal branches of this tree, so, for example, the value of 1,263 gene family gains on the basal branch of clade V will include gene families lost on the branch leading to the clade IV species. The first column of pie charts represents the gene family composition of each genome - the area of the circle is proportional to the predicted proteome size, and wedges represent the numbers of proteins predicted to be either singletons (that is, not members of any gene family), members of gene families common to all nine genomes, members of gene families present only in a single genome, and members of all other gene families. The second column of pie charts represents the genomic composition of the species with published genome sequences. All ingroup nodes had 100% bootstrap support.

**Figure 2:**
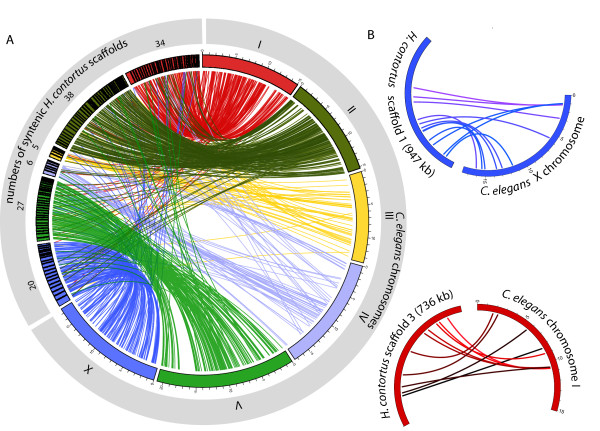
**One-to-one orthologs connecting *H. contortus *scaffolds and *C. elegans *chromosomes**. **(a) **All 130 *H. contortus *scaffolds with at least five one-to-one orthologs between the two species, with scaffolds ordered to maximize colinearity with the *C. elegans *genome. **(b) **The relative positions of one-to-one orthologs between the two largest *H. contortus *scaffolds and *C. elegans *chromosomes, demonstrating the lack of conservation of gene order within syntenic blocks.

We used extensive transcriptomic evidence from across the *H. contortus *lifecycle (Materials and methods; Table S3 in Additional file 1) to guide *de novo *gene model curation for protein-coding genes. We predict a similar number of protein-coding genes to the *C. elegans *genome (21,799 versus 20,532), but significantly lower gene density of 59 genes per Mb, with only 7% of the genome being protein coding, compared to 200 genes per Mb and a protein coding content of approximately 30% in the *C. elegans *genome (Figure 1). The average coding sequence length is similar in *H. contortus *and *C. elegans *(1,127 bp in *H. contortus *compared to 1,371 bp in *C. elegans*) but the average gene length is more than double in the parasite (6,564 bp compared to 3,010 bp in *C. elegans*). A closer look at a subset of 2,822 *H. contortus *and *C. elegans *one-to-one orthologs shows the discrepancy is explained by an expansion in both the number and length of introns in *H. contortus *(average of 10 introns per gene, average size 633 bp; relative to 6 introns per gene, average size 340 bp in *C. elegans*). An expansion in intronic sequence has also been reported in the closely related necromenic nematode *Pristionchus pacificus *but to a less dramatic extent (average of nine introns per gene, average size 309 bp) [26]. Over 80% of *H. contortus *and *C. elegans *one-to-one orthologs present on the same scaffold occur on the same *C. elegans *chromosome (Table S4 in Additional file 1), suggesting widespread conservation of synteny between the two species (Figure 2). However, gene order is generally poorly conserved, which is consistent with comparisons between *C. elegans *and related nematodes [27,31], but regions of conserved microsynteny are apparent and may prove useful in supporting orthology of divergent genes.

Reflecting this relatively expanded genome, approximately 29% of the draft genome assembly consists of repetitive sequence (compared to approximately 16% in *C. elegans*), including 2,434 copies of the characteristic HcRep element previously reported from a number of trichostrongylid species [14,32-34]. The repetitive sequence also includes representatives of many known repeat families in other nematodes, with approximately 6% of the genome derived from LINEs, 2.5% from long terminal repeat retrotransposons and 5% from DNA transposons, including TTAA-specific, Mariner-like and MuDR-superfamily elements, together with evidence of elements related to Tc1 and Tc4 of *C. elegans*. Despite belonging to similar families, *H. contortus *repeats represent independent expansions to those in other clade V nematodes, as repeat libraries from *H. contortus *show little similarity to genome sequence from other species, and vice versa. Our transcriptomic data suggest that active transposition is occurring, with evidence of expression for 4 out of 26 gene models annotated with transposase domains and 49 out of 482 reverse transcriptase domain-containing proteins. Expressed proteins appear to belong to a range of DNA transposon types, and to Gypsy-related and LINE retro-elements.

In *C. elegans*, around 17% of genes are in operons [35] - tightly linked clusters of two to eight genes, which are co-transcribed from the same promoter. The resulting polycistronic pre-mRNAs are resolved by *trans*-splicing with spliced leader (SL) SL1 and SL2 sequences. Most frequently, SL1 is *trans*-spliced to the first gene in an operon and downstream genes are SL2 *trans*-spliced. The structure (gene complement, order and orientation) of around 23% of *C. elegans *operons is conserved in the *H. contortus *genome. The structure of a further 10% of *C. elegans *operons appear to be partially conserved, where at least two orthologs are present on the same scaffold in the expected order and orientation, but one or more genes are in a different order, inverted or absent. Functional constraints are thought to conserve the intergenic distance in *C. elegans *operons to approximately 100 bp but genes in *H. contortus *operons are further apart: the average intergenic distance of genes with a conserved operon structure is 992 bp (median 621 bp, largest 8,329 bp), and the operon encoding ion channel subunits Hco-deg-2H and Hco-deg-3H has an intergenic distance of 2,342 bp [36].

Overall, SL1 *trans*-splicing was detected in 6,306 *H. contortus *genes and SL2 *trans*-splicing was detected in 578 genes. Of these, 318 *trans*-spliced genes were in the putative conserved operons identified above (Additional methods in Additional file 1). All 126 first genes in operons were *trans*-spliced to SL1 (SL2 *trans*-splicing was detected in five putative first genes, but examination of their loci suggests they are downstream genes in new operons in this species); 119 downstream genes were *trans*-spliced to SL1 and 73 were *trans*-spliced to SL2. If SL2 *trans*-splicing is the definitive criterion in identifying operons, the relatively low level of SL2 trans-splicing to downstream genes suggests that either operon function is less frequently conserved than operon structure in *H. contortus *or that undetected, divergent SL2 sequences are present. However, the relatively high frequency of SL1 *trans*-splicing in genes that are also SL2 *trans*-spliced (approximately 77%; 56 of 73 in conserved operons, 445 of 578 in all genes identified) suggests SL1 *trans*-splicing of downstream genes may also be relatively common in this species.

We employed two complementary approaches to global comparison of the *H. contortus *gene set, using the Inparanoid algorithm to look in detail at orthologs with *C. elegans *and *P. pacificus*, and OrthoMCL for a wider view of gene family evolution with other clade V nematodes. Of 5,937 orthology groups between *C. elegans *and *H. contortus*, 5,012 are one-to-one orthologs, while an additional 899 orthologs could be identified in *H. contortus *and *P. pacificus *but not *C. elegans*, suggesting they have been lost in the *C. elegans *lineage (Table S5 in Additional file 1). A number of orthology groups are significantly expanded in *H. contortus*, including a family of 180 *Haemonchus *paralogs to a single *C. elegans *gene that lacks any functional annotation (Table S6 in Additional file 1). Other expanded groups include genes with likely roles in parasitism, such as cysteine-rich secreted proteins, together with a set of helicase domains that include some with predicted signal peptides. Global analysis of the evolution of entire gene families (clusters of similar genes) across the clade V nematodes confirms this pattern of significant diversity within the clade (Figure 1), and allowed us to identify *H. contortus *genes lacking clear orthologs in other clade IV or V nematodes (Figure S2 and Table S8 in Additional file 1). This shows that the *Haemonchus*-specific proteome is enriched in genes encoding polypeptides involved in proteolysis, neurotransmission and carbohydrate metabolism, and in secreted proteins and those expressed in the cuticle. While some of these genes are explored in detail in the more focused analyses below, others may represent novel candidate genes involved in the host-parasite interface.

### Gene expression in parasite life-stages and gut

**Figure 3:**
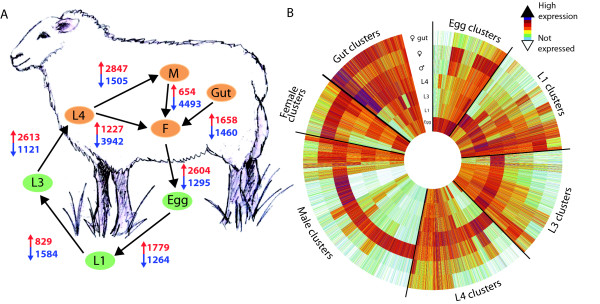
**Differential gene expression across the *H. contortus *lifecycle**. **(a) **Numbers of genes significantly up-regulated (red) and down-regulated (blue) between life-stages. **(b) **The expression profiles of genes determined to be differentially expressed between any pair of stages are shown in clusters ordered by the stage at which they are most highly expressed. The innermost circle shows normalized expression levels during the egg stage with further concentric circles showing expression in the L1, L3, L4 stages, followed by male worms, female worms and then female gut samples. Mean expression values were taken over three replicates and are shown on a log2 scale.

As *H. contortus *progresses through its lifecycle, it must adapt to different environments with differing food sources and energy requirements (Figure S1 in Additional file 1) and this is reflected in differential gene expression. RNA-seq was used to analyze gene expression in six parasite life-stages and the adult female gut. Samples were made in triplicate from independent batches of parasite material for every stage, allowing statistically robust comparison of relative gene expression between the stages (Materials and methods; Table S3 in Additional file 1). We found significant expression for 17,483 genes in total from the 6 life-stages studied; with between 13,962 (in L3) and 15,569 (in adult males) genes expressed in each stage. A total of 11,295 genes were significantly up- or down-regulated through the lifecycle (Figure 3 and Materials and methods) and we used annotation with Gene Ontology (GO) terms to investigate their broad functions. Metabolic enzyme expression throughout the parasite lifecycle was analyzed in more detail and will be discussed separately.

Genes up-regulated in the development from egg to the L1 stage include those associated with muscle development and motor activity, while up-regulated in the egg are genes associated with oxidoreductase activity, apoptosis, body morphogenesis and development, larval and embryo development, as well as DNA replication and chromosome organization. This pattern is consistent with the progression from a developing embryonated egg to a motile and actively feeding larval stage.

In the transition to the L3 stage, a decrease in the expression of genes associated with the myosin complex and motor activity and various metabolic processes are consistent with the nematode entering a quiescent state. Among the up-regulated genes there is an association with oxygen transport and heme binding. Oxidoreductase enzymes are over-represented and may reflect the increased need to detoxify a build up of endogenous waste, consistent with previous studies showing higher cytochrome P450 (CYP) activity in the *H. contortus *L3 than in L1 or adult stages [37]. CYPs have also been shown to be up-regulated in response to reduced food intake [38]. This is consistent with the significant increase in gluconeogenesis from the L1 to L3 - which is also increased in the *C. elegans *dauer [39] - and the up-regulation of acetyl-CoA metabolic process, likely to reflect metabolism of fat stores in the non-feeding L3. Genes associated with binding of cobalamin (vitamin B12) are also up-regulated. Cobalamin has been shown to be strongly concentrated and stored in the infective L3 of other gastrointestinal nematodes [40] and a ready supply may be required for rapid larval development after ingestion by the host.

The L4 is the first blood-feeding stage of *H. contortus*. The transition from the quiescent L3 stage to the motile L4 stage is reflected in significant up-regulation of many genes, including genes associated with motor activity, the myosin complex and locomotion as well as various metabolic processes. The binding of oxygen, lipids and sugars, possibly associated with active feeding, is also up-regulated, as are changes in the expression of genes linked to response to oxidative stress that may reflect the reactivation of the parasite from its dormant stage. A significant increase in the expression of genes associated with collagen and cuticle development and body morphogenesis, consistent with parasite growth, is also observed. Interestingly, heme-binding genes were both up- and down-regulated, perhaps reflecting an increase in heme load from blood feeding and a decrease in CYP activity.

Transition from the L4 to the adult stages is characterized by several changes. In the transition to the female stage, 1,658 genes are up-regulated and correlate with gender-specific development as well as embryogenesis (adult females contain oocytes and eggs at various developmental stages), such as genitalia development, embryo development, oogenesis, ovulation, germ-line cell cycle switching, meiosis regulation and regulation of vulval development. A significant increase in DNA replication processes is also apparent. Between the L4 and adult male stage, lower expression in the adult male of genes linked with body morphogenesis, molting, collagen and cuticle development, oxidoreductase activity, heme binding and response to oxidative stress were observed among the various alterations in expression. Increased expression of genes annotated with the GO term 'structure molecule activity' is due to up-regulation of a number of major sperm protein genes. These sex-specific gene expression patterns were confirmed by comparison between adult female and male parasites, and it is clear that many genes are highly enriched in male worms, being expressed only at low levels in other stages. The large set of genes highly expressed in both eggs and adult females were again apparent.

Finally, we investigated gene expression in the *H. contortus *intestine, the major organ of digestion and detoxification in the nematode, by comparing the female gut sample with the whole female worm. Consistent with data from *H. contortus *gut EST libraries [41], increased expression of genes with protein kinase, cysteine-type peptidase and cysteine-type peptidase inhibitor activities predominated. Genes associated with sugar and cobalamin binding were significantly up-regulated, as were genes associated with transport of cations, anions and oligopeptides. Oxidoreductase activity was also increased, consistent with the expression pattern of detoxification genes in *C. elegans *[42].

### Metabolic pathways and chokepoint analysis

Comparisons between *H. contortus *and the free-living nematodes revealed 22 enzyme classifications (ECs) that were restricted to the parasite (Table S9 in Additional file 1). While more detailed analysis is required, metabolism of amino acids and carbohydrates clearly differ between these two groups. For example, lysine 6-aminotransferase (EC 2.6.1.36) catalyzes lysine to glutamate, which can be further converted to α-ketoglutarate, an intermediate of the tricarboxylic acid cycle. Lysine 6-aminotransferase was previously considered restricted to prokaryotes; thus, its activity in *H. contortus *needs to be confirmed.

A summary of up- and down-regulated metabolic enzymes across all life-stages is shown in Table S10 in Additional file 1. The transition through eggs, L1, L3 and L4 showed a striking pattern: from L1 to L3, most enzyme classifications were down-regulated, including those involved in carbohydrate, lipid and energy metabolism, but many of these were up-regulated again in the transition to L4. This is consistent with the L3 being a stage in which development is arrested, analogous to the dauer larva in *C. elegans*. Further support for this comparison is the up-regulation of two enzymes that independently convert isocitrate to 2-oxo-glutarate, while most other parts of the tricarboxylic acid cycle are down-regulated. Furthermore, 2-oxo-glutarate is an entry metabolite into the ascorbate and aldarate metabolic pathway, which is implicated in increased lifespan in *Drosophila *[43]. The L4-to-male transition shows a decrease in lipid metabolism coupled with an increase in amino acid metabolism.

Metabolic chokepoints - reactions that uniquely consume or produce a metabolite - are enzymes that seem likely to be essential to the parasite and so may be potential targets for future drug development. Analysis of the *H. contortus *network revealed 362 chokepoint reactions, five of which passed additional criteria of lacking isoenzymes and being divergent from known human enzymes [44] (Table S11 in Additional file 1). Comparison of these against the Therapeutic Target Database revealed that two are known potential targets for anthelmintic drugs: trehalose-6-phosphatase (EC 3.1.3.12) is a member of the sucrose metabolism pathway, and is currently being researched as a potential drug target in the filarial nematode *B. malayi *[45], while dTDP-4-dehydrorhamnose 3,5-epimerase (EC 5.1.3.13) is currently of interest in *Mycobacterium tuberculosis *[46]. This validation of the approach justifies further analysis of the other three chokepoint enzymes identified.

### Neuromuscular drug targets

**Figure 4:**
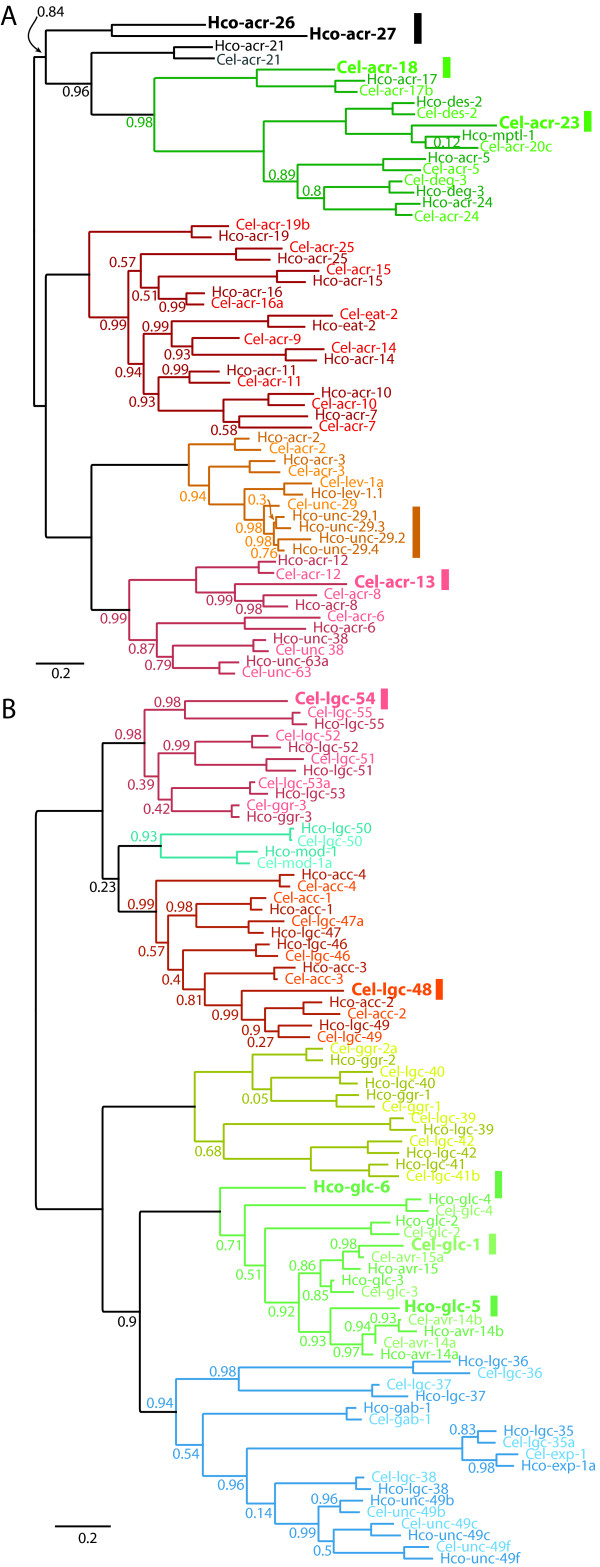
**Maximum-likelihood phylogenies of *H. contortus *and *C. elegans *ligand-gated ion channel families**. Figure shows unrooted trees for **(a) **cation channel and **(b) **anion channel genes from the two nematode species, with different colors representing each major clade of channels. Within clades, *C. elegans *genes are colored a lighter shade than *H. contortus *genes. Both trees show a general pattern of conserved one-to-one orthology, and colored bars indicate where this pattern is broken - by a duplication in *H. contortus *(with four copies of *unc-29*), and by loss of some genes - there are four *H. contortus *genes (*acr-26*, *acr-27*, *glc-5*, *glc-6*) without orthologs in *C. elegans*, and seven in *C. elegans *missing from *H. contortus *(*acr-13*/*lev-8*, *acr-18*, *acr-23*, *glc-1*, *lev-8*, *lgc-48*, *lgc-54*). All of these genes are shown in large bold type. A truncated ortholog of *C. elegans acr-9 *is present in *H. contortus *but is omitted from this phylogeny. Values near internal nodes are Shimodaira-Hasegawa test support values - with support values of 1.0 on all other nodes omitted for clarity.

In nematodes, the pentameric ligand-gated ion-channel (pLGIC) family is particularly numerous, with 64 members identified in *H. contortus *via homology with *Caenorhabditis *spp., *P. pacificus *and RNA-seq data (Figure 4). They are of great importance in parasitic nematodes because they are targets of the majority of the currently available anthelmintic drugs (as summarized in Table S12 in Additional file 1; the β-tubulin targets of the benzimidazole group have been described previously [47]).

The pLGICs regulate the flow of anions, typically chloride ions, or cations, including sodium and calcium, in response to an extracellular signal in the form of an activating ligand or change in pH. They are fundamental to synaptic transmission; interference with their normal function results in paralysis and death. Drugs that activate the anionic channels, such as the macrocyclic lactone ivermectin (IVM), typically inhibit neuronal transmission and muscle contraction. Those that activate the cationic channels, such as levamisole (LEV) and monepantal, stimulate neuronal transmission and typically induce muscle contraction. Here we present the most complete picture of these channels to date and show that, as expected, this parasite is very similar to *C. elegans*. This supports the use of the free-living worm as a functional model for the parasite nervous system. There are, however, some important differences, most significantly in glutamate signaling, which is sensitive to the macrocyclic lactones, and acetylcholine signaling, which is sensitive to LEV, as well as characteristic loss of some receptor genes associated with biogenic amine signaling.

The macrocyclic lactones, which include IVM and moxidectin, act at several different glutamate-gated chloride channels. Some of these are found in both *C. elegans *and *H. contortus*, but it is noteworthy that two *H. contortus *subunit genes, *glc-5 *and *glc-6*, encode glutamate-sensitive channels that are absent from *C. elegans*. It is likely these were lost from the rhabtidid lineage as homologous sequences can be detected in the genome of the close relative *P. pacificus*. Both of these subunits are targets for the macrocyclic lactones [48,49] and changes in either their sequence or expression have been associated with drug resistance in veterinary parasites [50]. The archetypal target of IVM, *Cel-glc-1*, appears to be a duplication of *avr-15*, specific to *C. elegans*. These differences may explain the difficulties encountered in understanding IVM's mode of action and resistance in parasitic nematodes by studying the model organism [51]. Most of the other anionic channel subunits in *H. contortus *have direct orthologs in *C. elegans *(Figure 4a). The only notable absences are *lgc-48 *and *lgc-54*. *lgc-48 *encodes an as yet uncharacterized member of the *acc-1 *acetylcholine gated chloride channels [52] that appears to have been lost from *H. contortus*, and *lgc-54 *encodes an uncharacterized member of the *C. elegans *biogenic amine-gated chloride channel family that may not be present in *H. contortus *[53].

The acetylcholine gated receptor cation channels are of particular interest as they are the targets of several anthelmintic drugs already in use (LEV, monepantel (MPTL) and derquantel) and are considered to be one of the most promising gene families for the identification of new drug targets. Comparison of the *H. contortus *gene family with those found in *C. elegans *is shown in Figure 4b. There is a one-to-one correspondence in *H. contortus *for subunits of the *acr-16 *clade [54], named for the homomeric nicotine-sensitive channel in *C. elegans*, and *acr-16 *expression is much higher in adult males than in other life-stages, as previously described for *C. elegans *[55]. The anthelmintic LEV targets receptors composed of α subunits of the *unc-38 *clade and non-α subunits from the *unc-29 *clade. In *C. elegans *three different alpha and two different non-alpha subunits combine to form a channel that responds to acetylcholine, strongly to LEV and only weakly to nicotine [56]. The α-type *acr-13 *(*lev-8*) gene, required in *C. elegans *for the LEV receptor, appears to have been lost in *H. contortus *as an ortholog of this gene is detectable in *P. pacificus*. The non-alpha gene *lev-1 *is present, but the signal peptide appears to have been lost [57]. A LEV receptor in *H. contortus *can be reconstituted without either of these, requiring only UNC-38, UNC-63 and UNC-29 as in *C. elegans *[57] and replacing ACR-13 with ACR-8. Most strikingly, the non-α subunit gene *unc-29 *in *C. elegans *corresponds to four paralogous copies in *H. contortus *[57]. A LEV-sensitive channel has been reconstituted containing the Hco-UNC-29.1 subunit [58]. The degree to which the other copies may have diverged in function is currently under investigation. The *deg-3 *clade [54] encodes subunits that form channels involved in chemotaxis, and is of particular interest as channels encoded by *des-2/deg-3 *and *acr-23 *in *C. elegans *are targeted by the relatively new anthelmintic MPTL, with *acr-23 *as the principal target of MPTL action *in vivo *[9]. The single subunit gene *mptl-1 *in *H. contortus *corresponds to the *acr-23*/*acr-20 *pair in *C. elegans*. Splice site mutations in the *mptl-1 *gene are associated with resistance to MPTL [36], which would suggest that *acr-23 *is functionally equivalent to *mptl-1*.

*H. contortus *possesses two acetylcholine gated receptor genes that are not present in *C. elegans*, *acr-26 *and *acr-27*, and these two genes seem to form a distinct clade, which suggests that they are likely to form receptors with a distinct pharmacology. Orthologs of *acr-26 *are found in many species of parasitic nematode and therefore they might make interesting targets for the development of novel cholinergic anthelmintics.

In summary, the majority of the pLGIC subunit genes in *C. elegans *have direct orthologs in *H. contortus*, although there is more variation in the repertoire of orphan family pLGICs (Additional results in Additional file 1). This supports the use of *C. elegans *as a model to study the neuromusculature of *H. contortus *in drug screens, but there are important differences in specific anthelminthic targets: for each of the anthelmintics IVM, LEV and MPTL, the drug targets in *H. contortus *are functionally different to the *C. elegans *model.

### Drug metabolism and efflux

Parasitic nematodes are armed with a large repertoire of inducible metabolizing enzymes and transporters to protect against environmental toxins. The nematode detoxification pathway can be divided into three main phases: modification, conjugation and excretion [59] involving the cytochrome P450s (CYPs) and the short-chain dehydrogenase/reductases (SDRs) in phase 1, the UDP-glucoronosyl transferases (UGTs) and the glutathione S-transferases (GSTs) in phase 2 and the ATP-binding cassette (ABC) transporters in phase 3. Little is known about the impact of the parasite detoxification system on anthelminthic efficacy or resistance [60], but a better understanding of these pathways will allow their role in anthelmintic resistance to be assessed and should also be informative in the design of new drugs and synergistic agents [61]. We have annotated a large number of modification and conjugation genes in the current assembly, identifying a total of 42 CYPs, 44 short-chain dehydrogenase/reductases, 34 UDP-glucoronosyl transferases and 28 glutathione S-transferases (Figure S5 in Additional file 1), but here we focus on the excretion transporters implicated in drug resistance.

Members of the ABC transporter family hydrolyze ATP and couple the energy released with the active transport of a wide range of compounds, including small organic molecules, lipids, proteins and metal ions. They are an essential component of many biological processes and are fundamental to the barrier between a nematode and its environment. These transporters consist of a basic structure of six transmembrane domains with an associated intracellular ATP binding motif. The functioning transporter complex requires two of these protein halves, but some members of this family are fusion proteins with 12 transmembrane domains and two ATP binding motifs. We find numerous differences in ABC transporter genes between *C. elegans *and *H. contortus *(Additional results and Figure S4 in Additional file 1), with a reduced complement of haf-transporters, including, for example, the loss of *haf-6*, which is essential for efficient RNA interference in *C. elegans *[62], a significant expansion of *ced-7*, which has an unknown function in amphid and phasmid sensory cells, and an extensive change in the repertoire of multidrug resistance protein genes. The P-glycoprotein (pgp) transporters are of particular interest as they have been implicated in resistance of *H. contortus *to IVM and other anthelmintics [49,63], and this family has undergone significant change compared to the free living *C. elegans*. In total, 10 P-glycoprotein genes have been identified in the *H. contortus *genome and knowledge of this full complement will now allow a more systematic analysis of the role of P-glycoproteins in resistance to IVM and other anthelmintics. A cluster of four *C. elegans *genes, *pgp-5*, *6*, *7 *and *8*, are not found in *H. contortus*. *Cel-pgp-3 *and *4 *as well as *Cel-pgp-12*, *13 *and *14 *represent gene duplication events corresponding to single genes in the parasite. *Cel-pgp-9 *corresponds to two paralogous copies in *H. contortus*, and in addition, two genes not present in *C. elegans*, related to *pgp-3 *and *pgp-11*, have been retained in *H. contortus*. These are named *pgp-16 *and *pgp-17*, respectively. Changes in the sequence or expression of *pgp-1*, *pgp-2 *and *pgp-9 *have been reported for IVM-resistant versus-susceptible isolates of *H. contortus *and in *pgp-9 *for resistant *Telodorsagia circumcincta *[50,64,65].

### Protease vaccine candidates

*H. contortus *is a voracious blood-feeder, with even modest infections of 1,000 worms generating losses of up to 50 ml of blood per day [66]. Cysteine, aspartic and metallo-proteases as well as aminopeptidases have been implicated in important aspects of parasite function, including hemoglobin digestion and anticoagulant activity, and these enzymes are also important vaccine candidates. Vaccination with gut extracts enriched for these activities can confer up to 75% reduction in worm burden and 90% reduction in egg output [67]. Protection is thought to result from the ingestion of host antibodies by the parasite during blood-feeding, which bind to gut antigens and disrupt function. Female parasites appear to be more affected than males, increasing the relative impact on egg output. Development of a commercial vaccine, however, requires identification and expression of specific proteases that, either singly or combined, induce protective immunity. Comparative genomics and transcriptome data can aid target selection by identifying potential functionally important proteases and those enriched in the gut of blood-feeding L4 and adult stages, suggesting a role in blood-feeding as well as accessibility to host antibodies.

**Figure 5:**
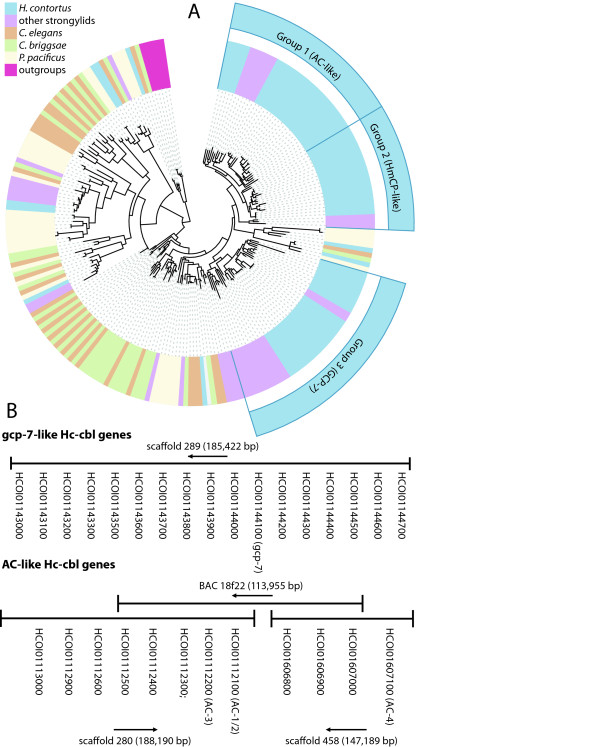
**(a) Maximum-likelihood phylogeny of cathepsin B (cbl) genes in *H. contortus *and related nematodes**. The *H. contortus *genome shows large, independent expansions of all three main classes of cbl genes: AC (anticoagulant)-like, GCP-7-like and Hmcp-like. **(b) **Many of the genes in two of these expansions occur as tandem arrays of duplicates on three scaffolds of the *H. contortus *genome. Two of these scaffolds are known to be linked, as eight of the genes occur on a previously completed bacterial artificial chromosome (BAC) sequence.

Cathepsin B protease (cbl) genes are part of an ordered hemoglobin degradation pathway, functioning after aspartic proteases (APRs) and upstream of metalloproteases (MEPs) and aminopeptidases [68,69]. Cathepsin B diversity may therefore be key in generating an array of substrates from ingested nutrients for efficient cleavage by downstream proteases and may be involved in the high blood digestion capacity of *H. contortus*. Indeed, *H. contortus *has a higher copy number (63 genes) of cathepsin B protease genes than related free-living nematodes, representing 80% of all cathepsin cysteine protease genes in the genome (Figure 5a). This large expansion of the cathepsin B family has resulted in *H. contortus *showing a greater diversity of cbl genes than known in any other parasitic nematode. Uniquely, members of each cbl type are arranged in tandem in the *H. contortus *genome (Figure 5b) and have the same genomic structure, suggesting that diversity has arisen through recent gene duplication. Reflecting this, most CBLs encoded in the *H. contortus *genome form large monophyletic groups distinct from *C. elegans *[70], hookworm [71] or other strongylid CBLs, suggesting that duplication and divergence has occurred separately following speciation. It is possible that gene amplification has occurred as a mechanism of increasing overall CBL expression associated with the need for *H. contortus *to digest the huge quantities of host blood it takes in during feeding. The cbl genes show increased expression in L4 and adult stages and many are significantly enriched in gut tissue, identifying these as potentially important control targets. These include both novel and previously identified cbl genes (*AC-4*, *gcp-7 *and *hmcp-2*) [72-74], while other cbl transcripts are significantly up-regulated in the L4 stage and in adult male worms, but not in the gut, suggesting a role in development and reproduction rather than feeding. The CBLs identified here contain a putative amino-terminal signal peptide and several have been identified in adult worm excretory-secretory products [75]. It has also been proposed that sequence variation of Hc-CBLs may confer antigenic diversity [76], and presentation to the host immune system through secretion may therefore drive the maintenance of the diversity of Hc-cbl genes. Three-dimensional modeling of the CBL repertoire and epitope mapping will clarify this issue.

Other cathepsin cysteine protease genes are not expanded - for example, a *cpr-6*-like single copy gene is highly conserved in a number of parasitic and free-living nematodes, suggesting a house-keeping role, and there is no expansion found for cathepsin L, F or Z genes in *H. contortus*. Furthermore, there is less expansion of genes encoding other vaccine candidates - H-gal-GP composed predominantly of pepsinogen-like aspartic and metallo proteases [77], and H11 aminopeptidases [78]. These are integral gut membrane proteins, considered hidden from the immune system during natural infection [67], and lower diversity should be advantageous in vaccination studies.

Significant expansion is, however, seen in the cathepsin aspartic protease family (Figure S6 in Additional file 1). Importantly, the genome data identify a novel, single-copy apr gene with 84% amino acid identity to APR-1 of the dog hookworm *Ancylostoma caninum *and that is significantly enriched in gut tissue. Vaccination with recombinant Ac-APR-1 significantly reduced fecal egg counts, worm burdens and anemia [79], warranting investigation of the protective potential of the *Haemonchus *APR-1-related protease. Other novel Hc-apr genes group phylogenetically with *C. elegans *asp genes (Figure S6 in Additional file 1) and are increased in the environmental L1 stage, indicating a developmental role, consistent with *C. elegans *data [80,81] and ruling these out as potential vaccine targets.

## Conclusions

*H. contortus *is the one of the most intensively researched parasitic nematode species and is the most established parasite species used in drug discovery, drug mode-of-action and drug resistance research. It is the first strongylid nematode to have its genome sequenced and the analysis presented here provides a first genomic insight into the biology of a gastro-intestinal strongylid and blood feeding parasitic nematode. It confirms the close relationship between *H. contortus *and *C. elegans *and provides a platform from which to apply the *C. elegans *biological knowledge to investigate the biology of this parasite for both basic and applied research. It also highlights specific differences between the species that will be important to inform researchers of those aspects of *C. elegans *biology that cannot be extrapolated. The genome sequence, gene models, transcriptome datasets and bioinformatic analyses provide a wealth of information on potential new drug and vaccine targets. They will also be an invaluable resource for the application of post-genomic technologies to this and other related strongylid parasites.

## Materials and methods

### Parasite material production and quality assurance

The MHco3(ISE) line is a *H. contortus *strain, susceptible to all the major anthelmintic classes, that has been passaged in the laboratory by serial infection for many years. It has been extensively phenotypically and genetically characterized previously [82]. The inbred MHco3(ISE).N1 strain was derived by a genetically validated single pair mating of an adult male and an adult female MHco3(ISE) worm using a method of direct transplantation into the abomasum of a recipient parasite-free sheep (described in [83]). The inbred MHco3(ISE).N1 strain was subsequently passaged by oral experimental infection of parasite-free sheep and parasite material was harvested using standard methods (see Additional methods in Additional file 1 for details). All parasite material used in this study was derived from the inbred *H. contortus *MHco3(ISE).N1 strain except for the female gut transcriptomic data, which were generated from the MHco3(ISE)strain. The MHco3(ISE) and MHco3(ISE).N1 strains were routinely monitored for genetic integrity at each passage, as was all parasite material derived from the strains, using a standard panel of microsatellite markers and a well established 'genetic fingerprinting' methodology [8,82]. Both the MHco3(ISE) and the MHco3(ISE).N1 are viably cryopreserved and available on request.

### Genome sequencing and assembly

We assembled a draft sequence of the *H. contortus *genome based on data from a mixture of sequencing technologies, including 21 × coverage of paired-end and shotgun reads from a Roche454SLX platform and approximately 163 × coverage of long- and short-insert paired-end libraries from an Illumina HiSeq (coverage based on a genome size of 370 Mb). Genomic and transcriptomic sequence data were generated using largely standard molecular biology methods (Additional methods in Additional file 1), except that whole-genome amplified material was used to generate sufficient material for a large-insert Illumina library from a single male worm, using a modified protocol. The genomic reads from each technology were initially assembled independently using assembly algorithms most suited to the typical coverage and read length of each, and scaffolds from each were then merged to form an initial set of scaffolds that were then improved by automated gap filling, followed by breaking and re-scaffolding using the paired-end data from both sequencing platforms.

### Protein coding gene prediction and functional annotation

After quality control and end-trimming, the transcriptome reads were mapped against the reference genome using TopHat software, v 2.0.6 (--mate-std-dev 50 -a 6 -i 10 -I 20000 --microexon-search --min-segment-intron 10 --max-segment-intron 50000) [84]. A reference dataset of 399 *H. contortus *protein encoding genes was manually curated from predictions of highly conserved genes using CEGMA (version 2.4) [24] and RNA-seq mapping. Of those, 347 were used to train Augustus [85] and 52 were used to independently evaluate the accuracy of the predictions. Final gene prediction (v2.0) was performed by Augustus using *H. contortus *specific parameters and RNA-seq, EST and polyA mappings as evidence hints generated by TopHat2 and PASA [86], respectively. Gene prediction accuracy was computed at the level of nucleotides, exons and complete genes on 52 manual curated gene models as described previously [87] and shown in Table S7 in Additional file 1). Functional annotation information was obtained from the interpro databases using interproscan v4.5 [88], GO [89] terms were annotated via interpro2GO and from the curated *C. elegans *annotation in Wormbase (release 235) [90] by assigning all GO terms shared by all *C. elegans *genes in a gene family to the *H. contortus *members of that family. Further functional insight was obtained by BLAST searches for similar genes in the GenBank nr database, and putative signal peptides were identified by SignalP [91]. To investigate *H. contortus *metabolism, a total of 828 ECs, covering 2,853 proteins, were assigned using KAAS [92], DETECT [93] and EFICAz [94]. Of these, 563 ECs covering 1,246 proteins were assigned to a metabolic pathway; the others are non-metabolic enzymes. Similar annotation efforts were carried out on *Caenorhabditis *species and *P. pacificus*.

### Gene expression and Gene Ontology analysis

The numbers of RNA-seq reads per gene model were counted using custom-made scripts making use of BEDtools and a gff file of the genome annotation, and based on the TopHat mapping described above. Analysis of gene expression was performed using the DESeq (v 1.8.1) package for Bioconductor [95]. Read coverage was normalized to estimate the effective library sizes for each library and negative binomial tests performed between pairs of sample triplicates, using dispersion estimates from the default approach, to obtain *P*-values for differential expression of each gene adjusted for false discovery rate using the Benjamini-Hochberg procedure for multi-testing [96]. Only genes with adjusted *P*-values ≤1e^-5 ^were retained. GO terms enriched in the set of differentially expressed genes in each comparison were identified using the 'weight01' algorithm of the TopGO (v 2.8.0) package for Bioconductor [97]. Only GO terms with *P *< 0.01 were considered for more detailed analysis. Expression data were drawn using Circos-0.62. All differentially expressed genes were clustered using MBCluster.seq with 50 clusters, and clusters then ordered based on which stage had the highest mean expression in that cluster.

### Data access

All sequences described in this paper have been submitted to the GenBank database, project ID PRJEB506. Sequence data are available at [98] and annotation has been submitted to GenBank and Wormbase. ENA accession numbers for all genomic and RNA-seq reads are listed in Tables S3, S13 and S14 in Additional file 1. The *H. contortus *genome assembly and functional annotation are available at [99].

## Abbreviations

ABC: ATP-binding cassette; APR: aspartic protease; bp: base pair; CBL: cathepsin B protease; CYP: cytochrome P450; EC: enzyme classification; EST: expressed sequence tag; GO: Gene Ontology; IVM: ivermectin; LEV: levamisole; LINE: long interspersed element; MPTL: monepantel; pLGIC: pentameric ligand-gated ion-channel; SL: spliced leader.

## Competing interests

The authors declare that they have no competing interests.

## Authors' contributions

MB, JAC, JSG and JDW designed research, which was coordinated by HB, MB, JSG and NH; DJB, ED, RL, AAM and NS maintained the parasite life cycle, performed the inbreeding crosses and contributed biological material; RL, ER and GIS prepared genomic DNA, RNA and transcriptome sequencing libraries; CB, RNB, DC, JAC, AG, MH, SLJ, IK, TK, KL, RL, AM, AJR, IJT, AW and JDW analyzed data; JAC, JSG and RL drafted the complete manuscript, with sections of text contributed by CB, RNB, DC, AG, SLJ, IK, TK, KL, AM, AJR, IJT, AW and JDW. All authors read and approved the final manuscript.

## Supplementary Material

Additional file 1 **Additional methods, results, figures and tables**. Click here for file

## References

[B1] Kaplan RM Drug resistance in nematodes of veterinary importance: a status report. Trends Parasitol 2004 14 477 481 10.1016/j.pt.2004.08.001 15363441

[B2] Kaplan RM Vidyashankar AN An inconvenient truth: global worming and anthelmintic resistance. Vet Parasitol 2012 14 70 78 10.1016/j.vetpar.2011.11.048 22154968

[B3] Prichard RK Basanez MG Boatin BA McCarthy JS Garcia HH Yang GJ Sripa B Lustigman S A research agenda for helminth diseases of humans: intervention for control and elimination. PLoS Negl Trop Dis 2012 14 e1549 10.1371/journal.pntd.0001549 22545163 PMC3335868

[B4] Gilleard JS The use of *Caenorhabditis elegans *in parasitic nematode research. Parasitology 2004 14 Suppl 1 S49 S70 16454899 10.1017/S003118200400647X

[B5] Geary TG Thompson DP *Caenorhabditis elegans*: how good a model for veterinary parasites? Vet Parasitol 2001 14 371 386 10.1016/S0304-4017(01)00562-3 11707307

[B6] Hashmi S Tawe W Lustigman S *Caenorhabditis elegans *and the study of gene function in parasites. Trends Parasitol 2001 14 387 393 10.1016/S1471-4922(01)01986-9 11685900

[B7] Gilleard JS Understanding anthelmintic resistance: the need for genomics and genetics. Int J Parasitol 2006 14 1227 1239 10.1016/j.ijpara.2006.06.010 16889782

[B8] Redman E Sargison N Whitelaw F Jackson F Morrison A Bartley DJ Gilleard JS Introgression of ivermectin resistance genes into a susceptible *Haemonchus contortus *strain by multiple backcrossing. PLoS Pathog 2012 14 e1002534 10.1371/journal.ppat.1002534 22359506 PMC3280990

[B9] Kaminsky R Ducray P Jung M Clover R Rufener L Bouvier J Weber SS Wenger A Wieland-Berghausen S Goebel T Gauvry N Pautrat F Skripsky T Froelich O Komoin-Oka C Westlund B Sluder A Mäser P A new class of anthelmintics effective against drug-resistant nematodes. Nature 2008 14 176 180 10.1038/nature06722 18337814

[B10] Taylor CM Martin J Rao RU Powell K Abubucker S Mitreva M Using existing drugs as leads for broad spectrum anthelmintics targeting protein kinases. PLoS Pathog 2013 14 e1003149 10.1371/journal.ppat.1003149 23459584 PMC3573124

[B11] Knox DP Redmond DL Newlands GF Skuce PJ Pettit D Smith WD The nature and prospects for gut membrane proteins as vaccine candidates for *Haemonchus contortus *and other ruminant trichostrongyloids. Int J Parasitol 2003 14 1129 1137 10.1016/S0020-7519(03)00167-X 13678629

[B12] LeJambre LF Windon RG Smith WD Vaccination against *Haemonchus contortus*: performance of native parasite gut membrane glycoproteins in Merino lambs grazing contaminated pasture. Vet Parasitol 2008 14 302 312 10.1016/j.vetpar.2008.01.032 18337013

[B13] Bethony JM Loukas A Hotez PJ Knox DP Vaccines against blood-feeding nematodes of humans and livestock. Parasitology 2006 14 Suppl S63 S79 17274849 10.1017/S0031182006001818

[B14] Laing R Hunt M Protasio AV Saunders G Mungall K Laing S Jackson F Quail M Beech R Berriman M Gilleard JS Annotation of two large contiguous regions from the *Haemonchus contortus *genome using RNA-seq and comparative analysis with *Caenorhabditis elegans*. PLoS One 2011 14 e23216 10.1371/journal.pone.0023216 21858033 PMC3156134

[B15] Blaxter ML De Ley P Garey JR Liu LX Scheldeman P Vierstraete A Vanfleteren JR Mackey LY Dorris M Frisse LM Vida JT Thomas WK A molecular evolutionary framework for the phylum Nematoda. Nature 1998 14 71 75 10.1038/32160 9510248

[B16] Taylor MA Coop RL Wall RL Veterinary Parasitology 2007 Oxford: Blackwell Publishing

[B17] Prichard R Genetic variability following selection of *Haemonchus contortus *with anthelmintics. Trends Parasitol 2001 14 445 453 10.1016/S1471-4922(01)01983-3 11530357

[B18] Waller PJ Rudby-Martin L Ljungstrom BL Rydzik A The epidemiology of abomasal nematodes of sheep in Sweden, with particular reference to over-winter survival strategies. Vet Parasitol 2004 14 207 220 10.1016/j.vetpar.2004.04.007 15219362

[B19] Hoberg EP Lichtenfels JR Gibbons L Phlyogeny for species of *Haemonchus *(Nematoda: Trichostrongyloidea): Considerations of their evolutionary history and global biogeography among *Camelidae *and *Pecora *(Artiodactyla). J Parasitol 2004 14 1085 1102 10.1645/GE-3309 15562609

[B20] Blouin MS Yowell CA Courtney CH Dame JB Host movement and the genetic structure of populations of parasitic nematodes. Genetics 1995 14 1007 1014 8582607 10.1093/genetics/141.3.1007 PMC1206824

[B21] Troell K Engstrom A Morrison DA Mattsson JG Hoglund J Global patterns reveal strong population structure in *Haemonchus contortus*, a nematode parasite of domesticated ruminants. Int J Parasitol 2006 14 1305 1316 10.1016/j.ijpara.2006.06.015 16950266

[B22] Gilleard JS Beech RN Population genetics of anthelmintic resistance in parasitic nematodes. Parasitology 2007 14 1133 1147 10.1017/S0031182007000066 17608973

[B23] Brasil BSAF Nunes RL Bastianetto E Drummond MG Carvalho DC Leite RC Molento MB Oliveira DAA Genetic diversity patterns of *Haemonchus placei *and *Haemonchus contortus *populations isolated from domestic ruminants in Brazil. Int J Parasitol 2012 14 469 479 10.1016/j.ijpara.2012.03.003 22787588

[B24] Leroy S Duperray C Morand S Flow cytometry for parasite nematode genome size measurement. Mol Biochem Parasitol 2003 14 91 93 10.1016/S0166-6851(03)00023-9 12706802

[B25] Parra G Bradnam K Korf I CEGMA: a pipeline to accurately annotate core genes in eukaryotic genomes. Bioinformatics 2007 14 1061 1067 10.1093/bioinformatics/btm071 17332020

[B26] Dieterich C Clifton SW Schuster LN Chinwalla A Delehaunty K Dinkelacker I Fulton L Fulton R Godfrey J Minx P Mitreva M Roeseler W Tian H Witte H Yang SP Wilson RK Sommer RJ The *Pristionchus pacificus *genome provides a unique perspective on nematode lifestyle and parasitism. Nat Genet 2008 14 1193 1198 10.1038/ng.227 18806794 PMC3816844

[B27] Ghedin E Wang S Spiro D Caler E Zhao Q Crabtree J Allen JE Delcher AL Guiliano DB Miranda-Saavedra D Angiuoli SV Creasy T Amedeo P Haas B El-Sayed NM Wortman JR Feldblyum T Tallon L Schatz M Shumway M Koo H Salzberg SL Schobel S Pertea M Pop M White O Barton GJ Carlow CK Crawford MJ Daub J Draft genome of the filarial nematode parasite *Brugia malayi*. Science 2007 14 1756 1760 10.1126/science.1145406 17885136 PMC2613796

[B28] Abad P Gouzy J Aury JM Castagnone-Sereno P Danchin EG Deleury E Perfus-Barbeoch L Anthouard V Artiguenave F Blok VC Caillaud MC Coutinho PM Dasilva C De Luca F Deau F Esquibet M Flutre T Goldstone JV Hamamouch N Hewezi T Jaillon O Jubin C Leonetti P Magliano M Maier TR Markov GV McVeigh P Pesole G Poulain J Robinson-Rechavi M Genome sequence of the metazoan plant-parasitic nematode *Meloidogyne incognita*. Nat Biotechnol 2008 14 909 915 10.1038/nbt.1482 18660804

[B29] Opperman CH Bird DM Williamson VM Rokhsar DS Burke M Cohn J Cromer J Diener S Gajan J Graham S Houfek TD Liu Q Mitros T Schaff J Schaffer R Scholl E Sosinski BR Thomas VP Windham E Sequence and genetic map of *Meloidogyne hapla*: A compact nematode genome for plant parasitism. Proc Natl Acad Sci USA 2008 14 14802 14807 10.1073/pnas.0805946105 18809916 PMC2547418

[B30] Kikuchi T Cotton JA Dalzell JJ Hasegawa K Kanzaki N McVeigh P Takanashi T Tsai IJ Assefa SA Cock PJ Otto TD Hunt M Reid AJ Sanchez-Flores A Tsuchihara K Yokoi T Larsson MC Miwa J Maule AG Sahashi N Jones JT Berriman M Genomic insights into the origin of parasitism in the emerging plant pathogen *Bursaphelenchus xylophilus*. PLoS Pathog 2011 14 e1002219 10.1371/journal.ppat.1002219 21909270 PMC3164644

[B31] Guiliano DB Hall N Jones SJ Clark LN Corton CH Barrell BG Blaxter ML Conservation of long-range synteny and microsynteny between the genomes of two distantly related nematodes. Genome Biol 2002 14 RESEARCH0057 12372145 10.1186/gb-2002-3-10-research0057 PMC134624

[B32] Hoekstra R Criado-Fornelio A Fakkeldij J Bergman J Roos MH Microsatellites of the parasitic nematode *Haemonchus contortus*: polymorphism and linkage with a direct repeat. Mol Biochem Parasitol 1997 14 97 107 10.1016/S0166-6851(97)00108-4 9297704

[B33] Johnson PCD Webster LMI Adam A Buckland R Dawson DA Keller LF Abundant variation in microsatellites of the parasitic nematode *Trichostrongylus tenuis *and linkage to a tandem repeat. Mol Biochem Parasitol 2006 14 210 218 10.1016/j.molbiopara.2006.04.011 16765463

[B34] Grillo V Jackson F Gilleard JS Characterisation of *Teladorsagia circumcincta *microsatellites and their development as population genetic markers. Mol Biochem Parasitol 2006 14 181 189 10.1016/j.molbiopara.2006.03.014 16687182

[B35] Allen MA Hillier LW Waterston RH Blumenthal T A global analysis of C. elegans trans-splicing. Genome Res 2011 14 255 264 10.1101/gr.113811.110 21177958 PMC3032929

[B36] Rufener L Maser P Roditi I Kaminsky R *Haemonchus contortus *Acetylcholine Receptors of the DEG-3 Subfamily and Their Role in Sensitivity to Monepantel. PLoS Pathog 2009 14 e1000380 10.1371/journal.ppat.1000380 19360096 PMC2662886

[B37] Kotze AC Cytochrome P450 monooxygenase activity in *Haemonchus contortus *(Nematoda). Int J Parasitol 1997 14 33 40 10.1016/S0020-7519(96)00161-0 9076527

[B38] Laing ST Ivens A Butler V Ravikumar SP Laing R Woods DJ Gilleard JS The Transcriptional Response of *Caenorhabditis elegans *to Ivermectin Exposure Identifies Novel Genes Involved in the Response to Reduced Food Intake. PLoS ONE 2012 14 e31367 10.1371/journal.pone.0031367 22348077 PMC3279368

[B39] Fuchs S Bundy J Davies S Viney J Swire J Leroi A A metabolic signature of long life in *Caenorhabditis elegans*. BMC Biol 2010 14 14 10.1186/1741-7007-8-14 20146810 PMC2829508

[B40] Weinstein PP Vitamin B12 changes in *Nippostrongylus brasiliensis *in its free-living and parasitic habitats with biochemical implications. J Parasitol 1996 14 1 6 8627475

[B41] Yin Y Martin J Abubucker S Scott AL McCarter JP Wilson RK Jasmer DP Mitreva M Intestinal transcriptomes of nematodes: comparison of the parasites *Ascaris suum *and *Haemonchus contortus *with the free-living *Caenorhabditis elegans*. PLoS Negl Trop Dis 2008 14 e269 10.1371/journal.pntd.0000269 18682827 PMC2483350

[B42] Chakrapani BP Kumar S Subramaniam JR Development and evaluation of an *in vivo *assay in *Caenorhabditis elegans *for screening of compounds for their effect on cytochrome P450 expression. J Biosci 2008 14 269 277 10.1007/s12038-008-0044-5 18535361

[B43] Lai CQ Parnell LD Lyman RF Ordovas JM Mackay TFC Candidate genes affecting *Drosophila *life span identified by integrating microarray gene expression analysis and QTL mapping. Mech Ageing Dev 2007 14 237 249 10.1016/j.mad.2006.12.003 17196240

[B44] Yeh I Hanekamp T Tsoka S Karp PD Altman RB Computational analysis of *Plasmodium falciparum *metabolism: organizing genomic information to facilitate drug discovery. Genome Res 2004 14 917 924 10.1101/gr.2050304 15078855 PMC479120

[B45] Kushwaha S Singh PK Rana AK Misra-Bhattacharya S Cloning, expression, purification and kinetics of trehalose-6-phosphate phosphatase of filarial parasite *Brugia malayi*. Acta Tropica 2011 14 151 159 10.1016/j.actatropica.2011.05.008 21658361

[B46] Kantardjieff KA Kim CY Naranjo C Waldo GS Lekin T Segelke BW Zemla A Park MS Thomas C *Mycobacterium tuberculosis *RmlC epimerase (Rv3465): a promising drug-target structure in the rhamnose pathway. Acta Crystallogr D Biol Crystallogr 2004 14 895 902 10.1107/S0907444904005323 15103135

[B47] Saunders GI Wasmuth JD Beech R Laing R Hunt M Naghra H Cotton JA Berriman M Britton C Gilleard JS Characterization and comparative analysis of the complete *Haemonchus contortus *β-tubulin gene family and implications for benzimidazole resistance in strongylid nematodes. Int J Parasitol 2013 14 465 475 10.1016/j.ijpara.2012.12.011 23416426

[B48] Forrester SG Prichard RK Dent JA Beech RN *Haemonchus contortus*: HcGluCla expressed in *Xenopus *oocytes forms a glutamate-gated ion channel that is activated by ibotenate and the antiparasitic drug ivermectin. Mol Biochem Parasitol 2003 14 115 121 10.1016/S0166-6851(03)00102-6 12798512

[B49] Glendinning SK Buckingham SD Sattelle DB Wonnacott S Wolstenholme AJ Glutamate-gated chloride channels of *Haemonchus contortus *restore drug sensitivity to ivermectin resistant *Caenorhabditis elegans*. PLoS ONE 2011 14 e22390 10.1371/journal.pone.0022390 21818319 PMC3144221

[B50] Blackhall WJ Pouliot JF Prichard RK Beech RN *Haemonchus contortus*: selection at a glutamate-gated chloride channel gene in ivermectin- and moxidectin-selected strains. Exp Parasitol 1998 14 42 48 10.1006/expr.1998.4316 9709029

[B51] Beech R Levitt N Cambos M Zhou S Forrester SG Association of ion-channel genotype and macrocyclic lactone sensitivity traits in *Haemonchus contortus*. Mol Biochem Parasitol 2010 14 74 80 10.1016/j.molbiopara.2010.02.004 20211658

[B52] Putrenko I Zakikhani M Dent JA A family of acetylcholine-gated chloride channel subunits in *Caenorhabditis elegans*. J Biol Chem 2005 14 6392 6398 10.1074/jbc.M412644200 15579462

[B53] De Graef J Claerebout E Vercruysse J Wolstenholme A Mitreva M Geldhof P Gene expression and mutation analysis of the parasite-specific glutamate-gated chloride channel GLC-6 in resistant *Cooperia oncophora *isolates following *in vivo *exposure to macrocyclic lactones. Int J Parasitol in press 10.1017/S0031182012001849 PMC3690601 23279803

[B54] Jones A Davis P Hodgkin J Sattelle D The nicotinic acetylcholine receptor gene family of the nematode *Caenorhabditis elegans*: an update on nomenclature. Invert Neurosci 2007 14 129 131 10.1007/s10158-007-0049-z 17503100 PMC2972647

[B55] Liu Y LeBeouf B Guo X Correa PA Gualberto DG Lints R Garcia LR A cholinergic-regulated circuit coordinates the maintenance and bi-stable states of a sensory-motor behavior during *Caenorhabditis elegans *male copulation. PLoS Genet 2011 14 e1001326 10.1371/journal.pgen.1001326 21423722 PMC3053324

[B56] Boulin T Gielen M Richmond JE Williams DC Paoletti P Bessereau JL Eight genes are required for functional reconstitution of the *Caenorhabditis elegans *levamisole-sensitive acetylcholine receptor. Proc Natl Acad Sci USA 2008 14 18590 18595 10.1073/pnas.0806933105 19020092 PMC2587545

[B57] Neveu C Charvet CL Fauvin A Cortet J Beech RN Cabaret J Genetic diversity of levamisole receptor subunits in parasitic nematode species and abbreviated transcripts associated with resistance. Pharmacogenet Genomics 2010 14 414 425 20531256 10.1097/FPC.0b013e328338ac8c

[B58] Boulin T Fauvin A Charvet CL Cortet J Cabaret J Bessereau JL Neveu C Functional reconstitution of *Haemonchus contortus *acetylcholine receptors in *Xenopus *oocytes provides mechanistic insights into levamisole resistance. Br J Pharmacol 2011 14 1421 1432 10.1111/j.1476-5381.2011.01420.x 21486278 PMC3221097

[B59] Lindblom TH Dodd AK Xenobiotic detoxification in the nematode *Caenorhabditis elegans*. J Exp Zool A Comp Exp Biol 2006 14 720 730 16902959 10.1002/jez.a.324 PMC2656347

[B60] Kotze AC Dobson RJ Chandler D Synergism of rotenone by piperonyl butoxide in *Haemonchus contortus *and *Trichostrongylus colubriformis in vitro*: potential for drug-synergism through inhibition of nematode oxidative detoxification pathways. Vet Parasitol 2006 14 275 282 10.1016/j.vetpar.2005.11.001 16325340

[B61] Jones BC Middleton DS Youdim K Lawton G, Witty DR Cytochrome P450 Metabolism and Inhibition: Analysis for Drug Discovery. Progress in Medicinal Chemistry 2009 14 Amsterdam: Elsevier 239 263 19328293 10.1016/S0079-6468(08)00206-3

[B62] Sundaram P Echalier B Han W Hull D Timmons L ATP-binding cassette transporters are required for efficient RNA interference in Caenorhabditis elegans. Mol Biol Cell 2006 14 3678 3688 10.1091/mbc.E06-03-0192 16723499 PMC1525249

[B63] Blackhall WJ Prichard RK Beech RN P-glycoprotein selection in strains of Haemonchus contortus resistant to benzimidazoles. Vet Parasitol 2008 14 101 107 10.1016/j.vetpar.2007.12.001 18241994

[B64] Williamson SM Storey B Howell S Harper KM Kaplan RM Wolstenholme AJ Candidate anthelmintic resistance-associated gene expression and sequence polymorphisms in a triple-resistant field isolate of *Haemonchus contortus*. Mol Biochem Parasitol 2011 14 99 105 10.1016/j.molbiopara.2011.09.003 21945142

[B65] Dicker AJ Nisbet AJ Skuce PJ Gene expression changes in a P-glycoprotein (Tci-pgp-9) putatively associated with ivermectin resistance in *Teladorsagia circumcincta*. Int J Parasitol 2011 14 935 942 10.1016/j.ijpara.2011.03.015 21683705

[B66] Clark C Kiesel G Goby C Measurements of blood loss caused by *Haemonchus contortus *infection in sheep. Am J Vet Res 1962 14 977 980 13879671

[B67] Newton SE Progress on vaccination against *Haemonchus contortus*. Int J Parasitol 1995 14 1281 1289 10.1016/0020-7519(95)00065-A 8635880

[B68] Gluzman IY Francis SE Oksman A Smith CE Duffin KL Goldberg DE Order and specificity of the *Plasmodium falciparum *hemoglobin degradation pathway. J Clin Invest 1994 14 1602 1608 10.1172/JCI117140 8163662 PMC294190

[B69] Williamson AL Lecchi P Turk BE Choe Y Hotez PJ McKerrow JH Cantley LC Sajid M Craik CS Loukas A A multi-enzyme cascade of hemoglobin proteolysis in the intestine of blood-feeding hookworms. J Biol Chem 2004 14 35950 35957 10.1074/jbc.M405842200 15199048

[B70] Larminie CG Johnstone IL Isolation and characterization of four developmentally regulated cathepsin B-like cysteine protease genes from the nematode *Caenorhabditis elegans*. DNA Cell Biol 1996 14 75 82 10.1089/dna.1996.15.75 8561899

[B71] Ranjit N Zhan B Stenzel DJ Mulvenna J Fujiwara R Hotez PJ Loukas A A family of cathepsin B cysteine proteases expressed in the gut of the human hookworm, *Necator americanus*. Mol Biochem Parasitol 2008 14 90 99 10.1016/j.molbiopara.2008.04.008 18501979

[B72] Pratt D Armes LG Hageman R Reynolds V Boisvenue RJ Cox GN Cloning and sequence comparisons of four distinct cysteine proteases expressed by *Haemonchus contortus *adult worms. Mol Biochem Parasitol 1992 14 209 218 10.1016/0166-6851(92)90071-Q 1574079

[B73] Rehman A Jasmer DP A tissue specific approach for analysis of membrane and secreted protein antigens from *Haemonchus contortus *gut and its application to diverse nematode species. Mol Biochem Parasitol 1998 14 55 68 10.1016/S0166-6851(98)00132-7 9879887

[B74] Skuce PJ Redmond DL Liddel S Stewart EM Newlands GFJ Smith W Knox D Molecular cloning and characterization of gut-derived cysteine proteinases associated with a host protective extract from *Haemonchus contortus*. Parasitology 1999 14 405 412 10.1017/S0031182099004813 10581619

[B75] Yatsuda AP Bakker N Krijgsveld J Knox DP Heck AJR de Vries E Identification of Secreted Cysteine Proteases from the Parasitic Nematode *Haemonchus contortus *Detected by Biotinylated Inhibitors. Infect Immun 2006 14 1989 1993 10.1128/IAI.74.3.1989-1993.2006 16495580 PMC1418636

[B76] Jasmer DP Mitreva MD McCarter JP mRNA sequences for *Haemonchus contortus *intestinal cathepsin B-like cysteine proteases display an extreme in abundance and diversity compared with other adult mammalian parasitic nematodes. Mol Biochem Parasitol 2004 14 297 305 10.1016/j.molbiopara.2004.06.010 15383300

[B77] Smith SK Smith WD Immunisation of sheep with an integral membrane glycoprotein complex of *Haemonchus contortus *and with its major polypeptide components. Res Vet Sci 1996 14 1 6 10.1016/S0034-5288(96)90121-6 8745246

[B78] Smith TS Munn EA Graham M Tavernor AS Greenwood CA Purification and evaluation of the integral membrane protein H11 as a protective antigen against *Haemonchus contortus*. Int J Parasitol 1993 14 271 280 10.1016/0020-7519(93)90150-W 8496010

[B79] Loukas A Bethony JM Mendez S Fujiwara RT Goud GN Ranjit N Zhan B Jones K Bottazzi ME Hotez PJ Vaccination with recombinant aspartic hemoglobinase reduces parasite load and blood loss after hookworm infection in dogs. PLoS Med 2005 14 e295 10.1371/journal.pmed.0020295 16231975 PMC1240050

[B80] Roy PJ Stuart JM Lund J Kim SK Chromosomal clustering of muscle-expressed genes in *Caenorhabditis elegans*. Nature 2002 14 975 979 12214599 10.1038/nature01012

[B81] Tcherepanova I Bhattacharyya L Rubin CS Freedman JH Aspartic proteases from the nematode *Caenorhabditis elegans*. Structural organization and developmental and cell-specific expression of asp-1. J Biol Chem 2000 14 26359 26369 10.1074/jbc.M000956200 10854422

[B82] Redman E Packard E Grillo V Smith J Jackson F Gilleard JS Microsatellite analysis reveals marked genetic differentiation between *Haemonchus contortus *laboratory isolates and provides a rapid system of genetic fingerprinting. Int J Parasitol 2008 14 111 122 10.1016/j.ijpara.2007.06.008 17727857

[B83] Sargison N Development of genetic crossing methods to identify genes associated with macrocyclic lactone resistance in the sheep nematode parasite, *Haemonchus contortus*. PhD Thesis 2009 University of Edinburgh

[B84] Trapnell C Pachter L Salzberg SL TopHat: discovering splice junctions with RNA-Seq. Bioinformatics 2009 14 1105 1111 10.1093/bioinformatics/btp120 19289445 PMC2672628

[B85] Stanke M Keller O Gunduz I Hayes A Waack S Morgenstern B AUGUSTUS: ab initio prediction of alternative transcripts. Nucleic Acids Res 2006 14 W435 W439 10.1093/nar/gkl200 16845043 PMC1538822

[B86] Haas B Salzberg S Zhu W Pertea M Allen J Orvis J White O Buell CR Wortman J Automated eukaryotic gene structure annotation using EVidenceModeler and the Program to Assemble Spliced Alignments. Genome Biol 2008 14 R7 10.1186/gb-2008-9-1-r7 18190707 PMC2395244

[B87] Guigó R Flicek P Abril JF Reymond A Lagarde J Denoeud F Antonarakis S Ashburner M Bajic VB Birney E Castelo R Eyras E Ucla C Gingeras TR Harrow J Hubbard T Lewis SE Reese MG EGASP: the human ENCODE Genome Annotation Assessment Project. Genome Biol 2006 14 S2.1 31 16925836 10.1186/gb-2006-7-s1-s2 PMC1810551

[B88] Zdobnov EM Apweiler R InterProScan - an integration platform for the signature-recognition methods in InterPro. Bioinformatics 2001 14 847 848 10.1093/bioinformatics/17.9.847 11590104

[B89] Ashburner M Ball CA Blake JA Botstein D Butler H Cherry JM Davis AP Dolinski K Dwight SS Eppig JT Harris MA Hill DP Issel-Tarver L Kasarskis A Lewis S Matese JC Richardson JE Ringwald M Rubin GM Sherlock G Gene Ontology: tool for the unification of biology. Nat Genet 2000 14 25 29 10.1038/75556 10802651 PMC3037419

[B90] Yook K Harris TW Bieri T Cabunoc A Chan J Chen WJ Davis P de la Cruz N Duong A Fang R Ganesan U Grove C Howe K Kadam S Kishore R Lee R Li Y Muller HM Nakamura C Nash B Ozersky P Paulini M Raciti D Rangarajan A Schindelman G Shi X Schwarz EM Ann Tuli M Van Auken K Wang D WormBase 2012: more genomes, more data, new website. Nucleic Acids Res 2012 14 D735 D741 10.1093/nar/gkr954 22067452 PMC3245152

[B91] Petersen TN Brunak S von Heijne G Nielsen H SignalP 4.0: discriminating signal peptides from transmembrane regions. Nat Methods 2011 14 785 786 10.1038/nmeth.1701 21959131

[B92] Moriya Y Itoh M Okuda S Yoshizawa AC Kanehisa M KAAS: an automatic genome annotation and pathway reconstruction server. Nucleic Acids Res 2007 14 W182 W185 10.1093/nar/gkm321 17526522 PMC1933193

[B93] Hung SS Wasmuth J Sanford C Parkinson J DETECT - A Density Estimation Tool for Enzyme Classification and its application to Plasmodium falciparum. Bioinformatics 2010 14 1690 1698 10.1093/bioinformatics/btq266 20513663

[B94] Arakaki A Huang Y Skolnick J EFICAz2: enzyme function inference by a combined approach enhanced by machine learning. BMC Bioinformatics 2009 14 107 10.1186/1471-2105-10-107 19361344 PMC2670841

[B95] Anders S Huber W Differential expression analysis for sequence count data. Genome Biol 2010 14 R106 10.1186/gb-2010-11-10-r106 20979621 PMC3218662

[B96] Benjamini Y Hochberg Y Controlling the false discovery rate: a practical and powerful approach to multiple testing. J R Stat Soc B (Methodological) 1995 14 289 300

[B97] Alexa A Rahnenfuhrer J topGO: Enrichment analysis for Gene Ontology, R package version 2.8.0. http://www.bioconductor.org/packages/2.12/bioc/html/topGO.html

[B98] Haemonchus contortus strain ISE/inbred ISE, whole genome shotgun sequencing project. http://www.ncbi.nlm.nih.gov/nuccore/CAVP000000000

[B99] *H. contortus *genome assembly and functional annotation. ftp://ftp.sanger.ac.uk/pub/pathogens/Haemonchus/contortus

