# Supplementary material for: The genome and transcriptome of Haemonchus contortus, a key model parasite for drug and vaccine discovery
Source: Genome Biol. 2013 Aug 28;14(8):R88. doi: 10.1186/gb-2013-14-8-r88 (PMC4054779; doi:10.1186/gb-2013-14-8-r88)
Supplement: Additional file 1 — Additional methods, results, figures and tables. [file gb-2013-14-8-r88-S1.PDF]

Supporting Information

Contents	Page
**SI Results**	
Testing for contamination and detecting heterozygosity	**2**
Inference of orthology	**2**
Repeat elements	**2**
Operons and spliced leader sequences	**3**
Neuromuscular drug targets	**3**
Drug metabolism and efflux	**4**
Evasion of host immunity	**5**
**SI Methods**	
Parasite material	**6**
Illumina sequencing	**6**
454 sequencing	**7**
Transcriptome sequencing	**7**
Genome assembly	**7**
Repeat analysis	**8**
Detecting heterozygosity and inference of orthology	**9**
Operon structure	**9**
Metabolic pathway and chokepoint analysis	**10**
Drug metabolism	**10**
Evasion of host immunity	**10**
**SI Figures**	
Figure S1. Detailed description of *H. contortus* lifecycle	**12**
Figure S2. Pattern of gene family sharing between three clade V nematode groups	**12**
Figure S3. Variation in metabolism across the *H. contortus* lifecycle	**13**
Figure S4. Maximum-likelihood phylogeny *of H. contortus* and *C. elegans* ABC transporters	**17**
Figure S5. Phylogenetic arrangement of drug metabolism genes in *H. contortus* and related nematodes	**18**
Figure S6. Phylogeny of cathepsin D aspartic protease (CTSD) genes in *H. contortus* and related nematodes	**19**
Figure S7. Base composition distribution of the *H. contortus* draft assembly	**19**
**SI Tables**	
Table S1. Genome and gene model statistics	**20**
Table S2. CEGMA analysis of assembly completeness	**21**
Table S3. RNA-seq libraries	**21**
Table S4. 130 scaffolds with more than five one-to-one *C. elegans* and *H. contortus* orthologs	**22**
Table S5. Summary of *H. contortus* and *C. elegans* gene orthology	**24**
Table S6. Species-specific expansions in *H. contortus* and *C. elegans* orthologs	**25**
Table S7. Accuracies of gene predictions by Augustus	**25**
Table S8. TopGO analysis of *H. contortus*-specific genes	**26**
Table S9. *H. contortus*-specific enzymes	**27**
Table S10. Comparison of enzyme expression in different life-stages	**27**
Table S11. Potential drug targets in *H. contortus* based on chokepoint analysis	**28**
Table S12. *H. contortus* LGIC genes that may be involved in anthelmintic action and resistance	**28**
Table S13. Illumina paired end sequence data for genome assembly	**29**
Table S14. Shotgun and paired end 454 sequence data for genome assembly	**29**
**References**	**29**

SI Results

Testing for contamination and detecting heterozygosity

H. contortus is an obligate parasite, so sequencing libraries could have significant contamination with environmental microorganisms or host material. The distribution of GC-content across the genome assembly shows no sign of any contamination (Figure S9), and comparison of our predicted protein set against the Genbank database showed few strong hits to microbe proteins. Another challenge of genome sequencing from non-model organisms is the high heterozygosity in populations, which can lead to different haplotypes assembling separately. Initial experiments with the recently published HaploMerger pipeline [1] suggest that around 18% of the current assembly may represent multiple copies of the same loci, where different haplotypes have assembled separately. However, we opted to not remove these scaffolds from the current assembly, as it is difficult to unambiguously resolve alleles from structural variation within a draft genome sequence. Based on analysis of allelic diversity (see SI Methods), 1120 genes (5%) are putatively allelic variants rather than duplications. suggesting that the presence of multiple haplotypes has had relatively little impact on our predicted gene set and so on downstream analyses.

Inference of orthology

In the *H. contortus* and *C. elegans* comparison, 5,937 orthologous clusters were identified, with 5,012 containing one-to-one orthologs (Table S5). The largest *H. contortus* family expansion has a 180:1 ratio, but no functional annotation is available for the *C. elegans* gene (Table S6). Large-scale expansions in *C. elegans* focus around the seven transmembrane-domain chemoreceptors. These genes are some of the largest families in *Caenorhabditis* species and have undergone species-specific expansions within the genus. The extreme ratios could be a consequence of *Caenorhabditid*-specific expansions, *Haemonchus* gene loss, or an over-conservative assembly for these loci. With respect to a global view of gene loss in *C. elegans*, 899 orthologous groups could be identified between *H. contortus* and *P. pacificus* to the exclusion of *C. elegans*.

Repeat elements

Characteristic repeat elements, HcRep, TecRep, TcRep and TteRep, have been sequenced in the *H. contortus*, *Teladorsagia circumcincta*, *Trichostrongylus colubriformis* and *Trichostrongylus tenuis* genomes respectively [2-5] and are associated with an upstream GT-rich tandem repeat. In trichostrongyloids, around half of all microsatellites are associated with this class of repeat element [4]. We have identified 2423 HcRep loci in the *H. contortus* genome, consisting of a GT-rich tandem repeat with one or more downstream copies of the HcRep repeat. The predicted secondary structures of HcRep, TcRep and TteRep loci have no conserved motifs and they have no homology with any known class of protein [4] so their mode of replication is unknown, but RNA-seq data shows HcRep elements are transcribed and we have previously identified seven concatenated HcRep sequences associated with a recent gene duplication breakpoint [6].

Operons and spliced leader sequences

In addition to the operons reported in the main text, an additional nine conserved operon loci with intergenic distances greater than 10 kb were identified but excluded from analysis on the likelihood they represent conservation of microsynteny only.

As well as looking at the pattern of trans-splicing across the genome, we also investigated the genomic repertoire of SL loci in *H. contortus*. In *C. elegans*, the 22 nt SL1 is donated by a 98 nt SL1 RNA, which is encoded with the 5S ribosomal RNA in 110 tandem 1 kB repeats on Chromosome V only. In the *H. contortus* draft genome, 185 scaffolds encode the published 22 nt *H. contortus* SL1 sequence (accession no. Z69630) at 100% identity, of which one scaffold encodes five identical copies, one scaffold encodes three identical copies and 20 scaffolds encode two identical copies of SL1. Other than the conserved SL1 region, there is little similarity with the *C. elegans* SL1 RNA sequence and the *H. contortus* 5S ribosomal RNA is encoded remotely from the SL, in tandem arrays elsewhere in the genome.

In *C. elegans*, there are 18 variant SL2 genes and they are dispersed throughout the genome. Five scaffolds encode the published 22 nt *H. contortus* SL2 sequence (accession no. AF2158360) at 100% identity, but only one of these scaffolds encodes multiple copies of SL2 and only three (of eight SL2-like sequences on the scaffold) are 100% identical. Four additional scaffolds can be identified encoding a single copy of a 22 nt SL2 sequence differing from the published sequence by 2-3 bp. Again there is little similarity with *C. elegans* SL2 RNA sequence beyond the spliced leader, but all novel SL2 sequences contained the characteristic motifs of nematode SL2 family members [7].

The SL RNA is assembled into a small nuclear ribonucleoprotein (snRNP) consisting of core Sm proteins plus two essential splicing factor proteins, previously isolated from *Ascaris suum* [8]. These splicing factor proteins are unnecessary for either *cis*-splicing or genic (alternate) *trans*-splicing and are absent from genomes that lack SL *trans*-splicing. Putative orthologs of genes encoding both proteins are present in the *H. contortus* genome. SL *trans*-splicing factors such as these, if essential in parasites but not present in their hosts, may provide novel targets for therapeutic intervention [8].

Neuromuscular drug targets

Failure to locate the *lgc-48* and *lgc-54* genes is not definitive proof that they are missing from *H. contortus*, as absence of a gene from a genome sequence that is not 100% complete is not proof that it does not exist.

Within the anionic channel subunit phylogeny, the *exp-1* and *lgc-50* subunits which form a distinct clade based on the sequence of the cys-loop and TM2 regions only [9], here form part of the *unc-49* clade of GABA-gated channel subunits based on subunit sequence that excludes only the highly variable intracellular loops and signal peptides. This pair is unusual in that they form a GABA-gated cationic, rather than anionic, channel. This placement is consistent with the idea that the ion-selectivity of a pre-existing GABA receptor switched from anionic to cationic [10].

Within the AChR cation channels, phylogenetic evidence suggests that the quadruplication of *unc-29* in *H. contortus* predates the speciation of *T. circumcincta, T. colubriformis* and *H. contortus* but post-

dates the split with *P. pacificus*. We note that the ortholog of *lev-1* in *H. contortus* in the current draft assembly is truncated at the amino terminus and lacks a signal peptide. Recently, a divergent AChR subunit gene, *acr-26*, has been identified in *H. contortus* [11] and a closely related subunit gene, *acr-27*, is also present only in *H. contortus*, and both these group closely with the ACR-21 subunit that is closely related to the vertebrate alpha-7 AChR subunit [12].

The orphan group of pLGICs has not been investigated in great detail and the biological function of most subunits in this clade is unknown. Overall, the comparison of *H. contortus* and *C. elegans* reveals a greater degree of change compared to the AChR and anionic channel families. One-to-one orthologs are identifiable for *lgc-5, -7, -9, -10, -20, -22, -25, -27* and *cup-4*. Divergent members have also been retained in *H. contortus* but lost from *C. elegans* including two genes related to the *lgc-5-8* group, one related to *cup-4* and three related to the *lgc-13-19* group. The *lgc-13-19* group itself is not present in *H. contortus* and sequence similarity suggests that these seven subunits may be derived from a single gene inherited by the ancestor of *C. elegans*. With the same level of similarity, it appears that *lgc-1* and *lgc-29* represent a *Caenorhabditis* specific duplication while *H. contortus* generated four copies independently from the same ancestral gene.

Drug metabolism and efflux

The *H. contortus* gene model predictions contain 42 CYPs (we have included 12 CYP genes manually curated from the 2008 supercontig database, which are not present in this draft assembly, for reasons we describe below), 44 SDRs, 28 GSTs and 34 UGTs (Figure S5). The detoxification enzyme gene families are apparently smaller in *H. contortus* than *C. elegans* and even more dramatic reduced gene complements have been reported in other parasitic nematodes including *Meloidogyne hapla, Meloidogyne incognita* and *Brugia malayi* [13-15]. An important caveat here is the rapid evolution of detoxifying gene families makes it possible that recent highly similar gene duplications, such as those reported in subfamilies of *C. elegans* CYPs, may be collapsed into single loci *in silico*. This will only be resolved with careful manual curation in future iterations of the assemblies together with detailed molecular genetic analysis.

The haf-transporters are composed of the basic TM-ABC unit, corresponding to the ABCB transporter type. They are typically found in the mitochondrial and endoplasmic reticulum membrane and are involved in the transport of metal ions and peptides. *H. contortus* shows a reduced complement of the haf-transporters with five genes, compared to nine in *C. elegans* (Figure S4). Genes *haf-1, 6, 7* and *8* are absent from *H. contortus* and while *haf-7* and *8* are not well characterized, *haf-1* is an inner mitochondrial membrane protein involved in the transport of peptides and *haf-6* is essential for efficient RNAi. The *haf-5* and *abtm-1* transporters are both present in *H. contortus* and are responsible for the protection from heavy metals and iron regulation. The pmp-transporters are also of the TM-ABC structure, corresponding to the ABCD type. They are found in the peroxisomal membrane and are involved in the protein traffic between the organelle and cytoplasm. *H. contortus* appears to have lost either *pmp-1* or *2* but possesses three paralogs of the *Cel-pmp-5* gene.

The P-glycoprotein (pgp) transporters are comprised of a duplication of the haf-transporter structure to give a TM-ABC-TM-ABC protein with twelve transmembrane domains and belong to the ABCB family. Defects in mammalian pgp lead to IVM sensitivity in both mice and dogs and led to the suggestion that they may play a role in resistance to IVM helminth parasites such as *H. contortus*.

Changes in pgp gene polymorphism and levels of gene expression have been associated with resistance to the macrocyclic lactones and benzimidazoles [16, 17].

The three abcf-transporter genes of the ABCF family lack any TM domains but possess the ATP binding motif of the ABC transporter family. They are involved in the regulation of protein synthesis and are highly conserved between *H. contortus* and *C. elegans*. The wht-transporter genes consist of the basic transporter structure, but with the TM and ATP binding domains reversed. They are members of the ABCG family to which the human breast cancer resistance gene ABCG2 belongs. Those genes that have been characterized are expressed in germline tissues and mutants have defects in sperm motility and fertilization. Of the seven wht genes in *C. elegans*, *wht-3* and *6* were not found in *H. contortus*, with paralogs of the *wht-5* transporter found twice (Figure S4). The abt-transporter genes of the ABCA family are expressed in pharyngeal and intestinal cells of *C. elegans* as well as some neuronal cells. They are involved in the transport of cholesterol and various other lipids. Orthologs of the genes *abt-1, 5* and *6* were not found in *H. contortus* but six paralogous copies of a gene related to the *ced-7* transporter member of this family were identified. The *ced-7* gene is involved in the process of phagocytosis of apoptotic cell corpses and functions as an active membrane transporter. It is expressed in the amphid and phasmid sensory cells involved in chemotaxis but it is not clear what its role in these cells is. The fact that so many copies of the gene are present in the parasite would suggest an expansion of whatever this function might be.

The multidrug resistance protein genes, mrp, are of the ABCC family of transporters with a set of five TM domains, followed by two sets of six TM domains with their associated ATP binding motif regions. Their primary function is the transport of small organic molecules, conjugated with glutathione. The *mrp-3, 4, 7* and *8* genes have direct orthologs in *H. contortus* and *C. elegans*, but *mrp-5* and *6* have no detectable ortholog in *H. contortus* and *mrp-1* and *2* represent a gene duplication in *C. elegans* of a gene found once in *H. contortus* (Figure S4). In addition to these, *H. contortus* has retained a divergent member of the family that appears to have been lost from the *C. elegans* lineage.

Evasion of host immunity

A parasitic organism possesses a wide variety of tools to evade the immunity of its host. One approach is to mimic the proteins of the host immune system to regulate it in favour of the parasite. Another is to actively degrade any immediate threat in the physical vicinity [18]. In our search for such mechanisms, we surveyed the genome of *H. contortus* for homologues of the mammalian immune proteins. As described in the main text, *H. contortus* has undergone a large expansion in the cathepsin-like protease gene family. This family of proteins is commonly associated with infectivity, tissue migration and suppression of the host immunity. In addition to the expansion of cathepsin B proteases (Figure 5), we also discovered significant expansion of cathepsin D-like aspartic proteases (CTSD) in *H. contortus*. A phylogenetic analysis of CTSD has separated most of the sequences in six distinct clusters (Figure S6). Additionally, we have observed that the sequences from *P. pacificus* behave similarly, organizing in two clusters. Notably, the sequences in each *H. contortus* group appear to have similar expression patterns, as determined by RNA-seq. For example, sequences in group 2 are more highly expressed in L4 and male, while group 3.1 sequences are enriched in female and group 4 sequences are more highly expressed in L1. The latter is consistent with *C. elegans asp-1* expression [19] and rules out this group as potential vaccine targets. This might suggest that

proteases with particular substrate specificities are expressed at specific stages of the lifecycle, and possible play a developmental role.

Despite our expectations, we have not observed expansions in the core regulators of the immune system, such as the interleukins or TGF-β family of proteins [20].

SI Methods

Parasite material

All experimental procedures described in this manuscript were examined and approved by the Moredun Research Institute Experiments and Ethics Committee and were conducted under the legislation of a British Home Office license in accordance with the Animals (Scientific Procedures) Act of 1986. The Home Office license number is PPL 60/3899 and experimental ID for this study is E34/09.

Four to nine month old lambs that had been reared and maintained indoors under parasite-free conditions were infected by oral administration of 5000 *H. contortus* L3 to generate the appropriate life-stages for this study. The exception was one lamb infected with 150,000 L3 to generate the L4 stage. All samples were prepared from the inbred *H. contortus* MHco3(ISE).N1 isolate, other than the adult female gut libraries, which were prepared from the MHco3(ISE) isolate[21].

Parasite material was retrieved using standard techniques described in Jackson and Hoste [22]. Briefly, faeces were collected from mono-specifically infected sheep and *H. contortus* eggs were recovered with differential sieving and salt flotation. Half of the eggs were immediately snap-frozen in liquid nitrogen for RNA isolation and the remainder were incubated at 22°C for 24-48 hours to generate first stage larvae (L1), which were Baermann filtered, counted then snap-frozen in liquid nitrogen. Faeces were incubated at 22 °C for 14 days to culture third stage larvae (L3), which were retrieved by Baermann filtering, counted and snap-frozen in liquid nitrogen. Adult worms were harvested 28 days post infection from the abomasa of sheep at post mortem using an agar/mesh flotation method. The worms were rinsed, sexed, counted and snap-frozen in liquid nitrogen. Fourth stage larvae (L4) were isolated from the abomasum of a donor sheep on post mortem, at seven days post infection. The abomasum was suspended in physiological saline (0.85% NaCl w/v) at 38°C for 3-5 hours to digest the mucosa and release the L4, which were rinsed and snap-frozen in liquid nitrogen. The *H. contortus* gut was dissected from adult female worms using a protocol described by Rehman and Jasmer [23] and placed directly into Trizol (Invitrogen, 15596-026).

Illumina sequencing

Illumina libraries originated from a single *H. contortus* MHco3(ISE).N1 adult male worm. The worm was cut into three pieces and 48 ng genomic DNA (gDNA) obtained using the Agencourt Genfind v2 kit following the manufacturer's instructions, apart from digestion with proteinase K at 65°C with DTT. After RNase A treatment, 40 ng gDNA was used directly for preparation of an amplification-free 400 bp paired end Illumina library using a protocol based on a previously described method [24] but using Agencourt AMPure XP beads for sample clean up and size selection. DNA was precipitated onto beads after each enzymatic stage with a 20% polyethylene glycol 6000 and 2.5M sodium chloride solution. Beads were not separated from the sample throughout the process until after the adapter ligation stage and then fresh beads were used for size selection.

5 ng gDNA from the single male worm was whole genome amplified (WGA) using BioHelix Rapisome pWGA kit following the manufacturer's instructions except for a two hour incubation at 37°C. The WGA DNA was used to generate a 3 kb mate pair library using a modified SOLiD 5500 protocol adapted for Illumina sequencing [25]. Further details are in Table S13.

Libraries were denatured with 0.1M sodium hydroxide and diluted to 6pM in a hybridisation buffer to allow the template strands to hybridise to adapters attached to the flow cell surface. Cluster amplification was performed on the Illumina cBOT using the V4 cluster generation kit following the manufacturer's protocol and then a SYBR Green QC was performed to measure cluster density and determine whether to pass or fail the flow cell for sequencing, followed by linearization, blocking and hybridization of the R1 sequencing primer. The hybridized flow cells were loaded onto the Illumina HiSeq for 100 cycles of sequencing-by-synthesis using the V5 SBS sequencing kit then *in situ* the linearization, blocking and hybridization step was repeated to regenerate clusters, release the second strand for sequencing and to hybridise the R2 sequencing primer followed by another 100 cycles of sequencing to produce paired end reads. These steps were performed using proprietary reagents according to the manufacturer's recommended protocol [26]. Data was analysed from the Illumina HiSeq sequencing machines using the RTA1.8 analysis pipelines.

454 sequencing

gDNA was prepared from *H. contortus* MHco3(ISE).N1 adult worms using a standard phenol/chloroform protocol and ethanol precipitation. Samples were treated with RNase A to remove contaminating RNA. Microsatellite markers were used to confirm isolate identity [27]. The resulting gDNA was used to produce paired end (3 kb, 8 kb and 20 kb) and shotgun 454 libraries (Table S14) using standard Roche protocols [28] and sequenced using the 454 Life Sciences GS-20 and GS-FLX sequencer (Roche).

Transcriptome sequencing

Total RNA was isolated from all *H. contortus* life-stages, other than the L2, and the worm gut using a standard Trizol (Invitrogen, 15596-026) protocol and samples were run on a Bioanalyser 2100 (Agilent) to assess quality and quantity. Illumina transcriptome libraries (Table S3) were prepared using a protocol described previously [6]. All libraries were made in triplicate, using (per replicate) ~200,000 eggs, ~65,000 L1, ~250,000 L3, ~30 L4, 20 adult males, 20 adult females, and 20 female guts. The transcriptome libraries were run on Illumina HiSeq sequencing machines, as described for Illumina sequencing of the genome above, to generate paired end 100 bp reads.

Genome assembly

Our genomic sequence data consisted of 14x coverage of Roche 454 shotgun sequence reads, 7x coverage of 454 paired-end reads, 97x coverage of Illumina short-insert and 66x coverage of Illumina large-insert reads (based on a genome size of 370 Mb). All 454 data was assembled using Celera assembler version 7.0 [29], using the *mer* overlapper with *k*-mer size 31 and parameters *utgErrorRate* 0.03, *utgErrorLimit* 2.5, *ovlErrorRate* 0.06, *cnsErrorRate* 0.10, *cgwErrorRate* 0.10. Scaffolds smaller than 500 bp from this assembly were discarded, producing an initial 454 assembly of 53,040 scaffolds with a total length of 407.9 Mb, scaffold N50 of 36.4 Kb. All Illumina data was assembled using an in-house pipeline. Here, reads were first corrected using the read correction step

from SGA v0.9.7 [30], with parameters *"-f 5 -q 5 -p 1 -m 71 -p 1"* for the pre-processing step and parameters *"-k 41 --learn –discard"* for the correction step, followed by filtering low-quality reads with parameters *"-d 256 -x 2"*. SGA v0.9.7 was then used to generate an initial assembly using *"-m 77 -d 0.4 -g 0.1 -r 10 -l 200"*. This draft assembly was then used to calculate the distribution of *k*-mers for all odd values of *k* between 41 and 81, using the *suffixerator* and *tallymer* command from GenomeTools v.1.3.7 [31]. The *k*-mer length for which the maximum number of unique *k*-mers were present in the SGA assembly was then used as the *k*-mer setting for de Bruijn graph construction in a second assembly with Velvet v1.2.03 [32], using the corrected reads from SGA with flags *"-cov_cutoff auto -exp_cov auto -very_clean yes -min_contig_lgth 500"*, and insert lengths for Illumina libraries as listed in Table S1. This Velvet assembly was then treated as the initial assembly for Illumina data. The SGA assembly was used only to parameterise the Velvet step, and was discarded after this step. In each case, the detailed parameters above are the result of extensive experimentation with data from a range of parasitic nematodes. The initial Illumina assembly consisted of 21,476 scaffolds over 500 bp in length, totalling 348.9 Mb, scaffold N50 of 38.4 Kb.

Scaffolds from both Illumina and 454 assemblies were merged using GAA version 1.0 [33], using default scoring parameters. Our Illumina sequence data is from a single male worm, and so will contain only a pair of haplotypes for each chromosome, while the 454 data is from pools of mixed gender adult worms. As *H. contortus* populations are highly diverse, we attempted to avoid producing an assembly with many different haplotype sequences by using the Illumina assembly as a 'target' in GAA, and 454 as 'query', so that the 454 data is only retained where it can extend or join illumina scaffolds. This process was followed by automated gap-filling and contig extension using GapFiller_v1-10 and IMAGE [34, 35] with the short-insert Illumina data. Following this process, the assembly scaffolds were re-scaffolded using SSPACE BASIC v2.0 [36] to allow us to make scaffolding joins based on extended or merged sequence from the previous two steps. SSPACE was run with no contig extension, with 31 bp required between merged contigs, as an iterative procedure, using each long insert library in order of decreasing fragment size. For each library, SSPACE was run nine times, with the number of links needed between contigs to scaffold being 60, 30, 20, 10, 10, 7, 7, 5, 5, and 5, to allow the strongest scaffolding links to be formed before weaker scaffolding evidence is included. Finally, scaffolds and contigs within the assembly were broken wherever there was no coverage of fragments from the long-insert sequencing libraries (libraries with > 3 kb fragment size) to minimize the chance of mis-assembly.

Repeat analysis

Repeats were identified using two complementary approaches: Transposable elements in the assembly were identified using three approaches: RepeatModeler (version open-1.0.5) was used to detect repetitive DNA sequences de novo, LTRharvest from GenomeTools v1.5.1 was used to specifically identify long terminal repeat retrotransposons, and transposonPSI (version 08222010) used to identify known transposable elements. Consensus repeat sequences from all three approaches were clustered using usearch v5.0.151 with 80% identity to generate a non-redundant library of 2,604 consensus sequences for repeats, of which 114 were removed as showing similarity to known protein-coding genes or ncRNAs from wormbase release WS236. RepeatMasker (v3.2.8) was used to calculate the distribution of each repeat and its abundance.

Detecting heterozygosity and inference of orthology

To distinguish between allelic and truly duplicated genes, an approach modified from the method described by Barriere and colleagues [37] was used. Briefly, *H. contortus* genes were considered allelic if: they were in an inparalogous relationship with a single *C. elegans* gene as determined by Inparanoid [36], they were each other's best reciprocal BLAST hit, the length of the alignment was at least 60% of the length of the query sequence, the percent identity of the alignment met a size-dependent threshold, their shared amino acid identity was at least 90%, and they were located on different scaffolds of the assembly. An assumption was that no more two alleles were present for any gene. The Inparanoid algorithm was used to determined orthologous and paralogous relationships with *C. elegans* and *P. pacificus* [38]. Two searches were undertaken, one with *P. pacificus* as the outgroup as per the accepted species phylogeny, and a second with *C. elegans* set as the outgroup. This second search serves to identify genes orthologous between *H. contortus* and *P. pacificus* that have been lost in the *Caenorhabditis* lineage. For each of the candidate allelic pairs the copy on the longer scaffold was kept as the true gene, the other copy was discarded.

Predicted *H. contortus* genes, together with genes of other published clade V nematodes (*C. elegans*, *C. briggsae, C. japonicum, C. remanei, C. brenneri* and *P. pacificus*), together with two representative outgroups from clade IV (*M. hapla* and *Bursaphelenchus xylophilus*) were clustered into gene families using OrthoMCL v.2.0 [39]. Phylogenetic analysis of gene family evolution was based on a maximum-likelihood phylogeny based on analysis of concatenated amino acid sequences of 610 OrthoMCL gene families that were single-copy and present in all nine genomes analyses. Sequences were aligned before concatenation with Mafft v.6.857. [40] using '--auto', and the alignments cleaned using trimAl v.1.4 [41] with flag '-automated1'. After concatenation, phylogenetic analysis used RAxML v7.2.8 [42] using the best-fitting empirical model (minimum AIC) of amino-acid substitution and including estimated base frequencies, proportion of invariant sites and alpha parameter of a gamma distribution of rate variation across alignment columns, and using RAxML to estimate the maximum likelihood phylogeny for the concatenated alignment using default settings, and for 1,000 bootstrap resampling replicates. The birth and death of gene families on this phylogeny was inferred using Dollo parsimony using the Dollop program from v3.69 of the Phylip package [43].

Operon structure

Protein sequences for *C. elegans* genes in operons [44] were retrieved from Wormbase in eight batches based on their position in an operon (first gene to eighth gene). These were used to identify putative orthologs in the *H. contortus* gene models and candidate operons were assembled based on shared locus, orientation and conserved *C. elegans* gene position.

Reads containing SL sequences were extracted, trimmed to remove the SL and mapped to the genome as described previously [6]. Gene models lying within 1000 bp upstream or downstream of SL-trimmed reads were identified and compared to the dataset of putative conserved operons. The loci of all SL2 trans-spliced genes not in this dataset were considered to be candidate novel operons.

Metabolic pathway and chokepoint analysis

The Enzyme Classification (EC) system was used to annotate *H. contortus* proteins with enzymatic activities. The combined predictions from three software were used: KEGG automatic annotation server (KAAS) [45], DETECT [46] and EFICAz [47].

Chokepoint analyses followed the approach described by Yeh and colleagues [48]. Briefly, a chokepoint reaction is one that either uniquely consumes or produces a specific metabolite. From this list of reactions, those catalysed by isoenzymes and by enzymes whose proteins shared sequence similarity with human or mouse proteins (e<0.001) were removed. Those enzymes that catalysed this reduced set of chokepoint reactions were searched against the Therapeutic Target Database to identify known chemical inhibitors [49].

For the life-stage changes in metabolism, adjusted p-values were used. A two-fold change in expression was required to be classed as differentially expressed. Pathway maps were generated using the iPath visualisation tool [50].

Drug metabolism

Searches for detoxification enzymes were seeded with sequences from *C. elegans* (Wormbase, WS234), which were annotated as CYPs, SDRs, GTSs or UGTs and contained the appropriate Pfam domain: CYP - PF0067; SDR - PF00106; GST - PF00043, PF13410 and/or PF02798; UGT - PF00201. Identification of potential homologues in *H. contortus* and *P. pacificus* used a combination of fuzzy-reciprocal BLASTP and HMMer (v3.0) [51] searches. The MEME motif-finding tool (v. 4.5.0) [52] along with MAST search programme [53] was used to help to identify any genes where homology could be confounded by an error in the gene model.

For each enzyme class, sequences of the three species were aligned using the MAFFT method [40] and the L-INS-i algorithm implemented in Geneious ver. 6.0.5 (Biomatters Ltd). The alignments were manually curated to remove very short sequences and likely allelic pairs. Maximum Likelihood (ML) trees for the three gene families were then estimated using the software PhyML ver. 3.0 [54]. The WAG substitution model [55] with rate heterogeneity across sites was used. Branch supports were assessed using the approximate likelihood ratio test (aLRT) with Shimodaira-Hasegawa-like (SH-like) supports [56].

Evasion of host immunity

The ImmPort database [57] was used as a reference source of the human immune system [58]. The list of orthologs in *Mus musculus* and *Bos taurus* from the database was used to construct a representation of the mammalian immune system. For each gene with a unique ENSEMBL identifier, the corresponding protein sequences, including transcript variants, were gathered from the three organisms. The protein models for each gene group were constructed using HMMER3 [51]. These models were then employed to search the genome of *H. contortus* and three free living nematode species: *C. elegans, C. briggsae* and *P. pacificus*. The sequences with e-values of under 1e-6 were selected for downstream analyses. Copy numbers were counted for each of the four nematode species and those with a higher number in *H. contortus* were selected. The sequences were aligned using MAFFT's E-INS-i algorithm [59] and PhyML was used to construct the phylogenetic trees, using

the Le and Gascuel [60] amino acid replacement model. The trees were bootstrapped 100 times for support.

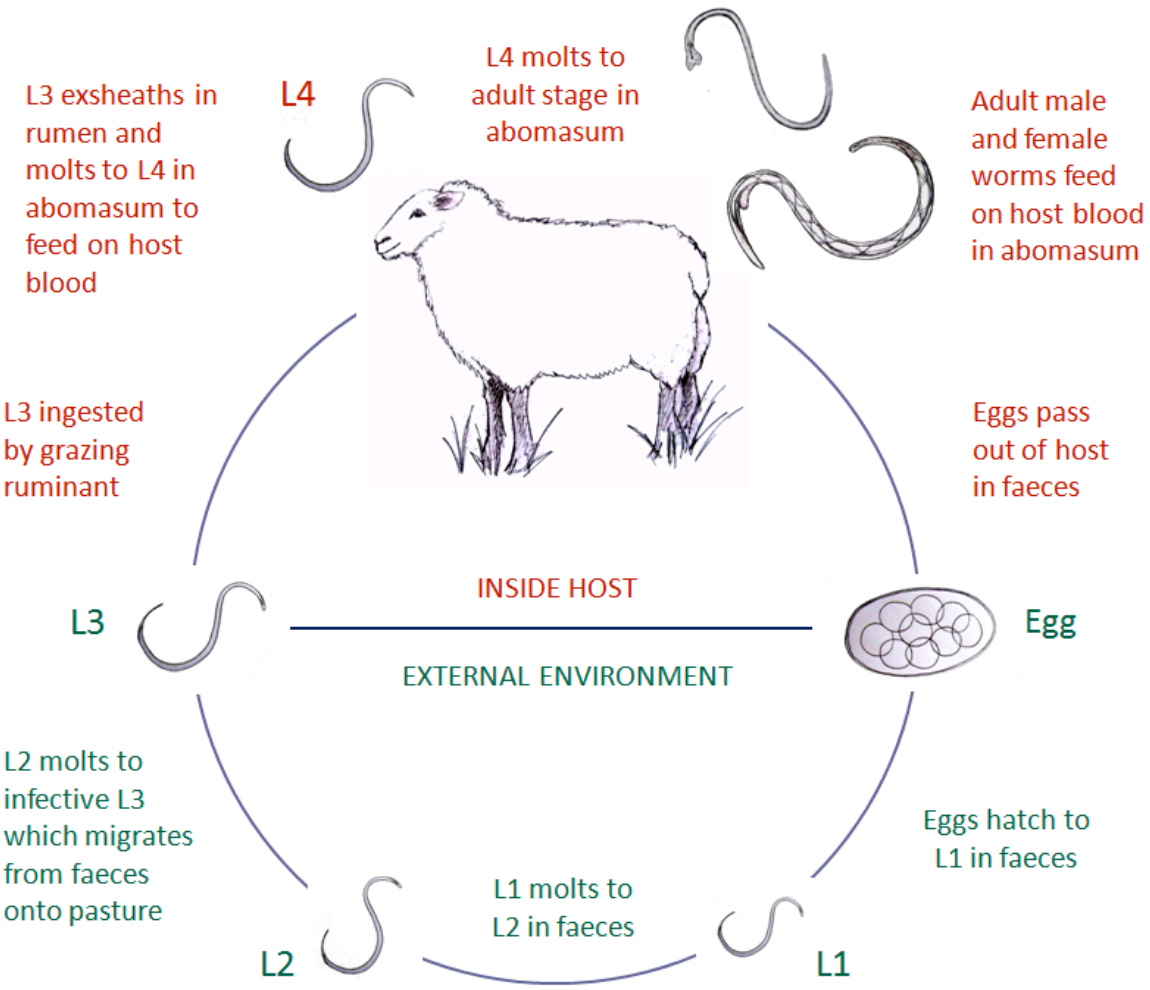

Figure S1. Detailed description of *H. contortus* lifecycle. Parasite life-stages within the host are coloured red and life-stages in the external environment are coloured green. The pre-patent period is two to three weeks.

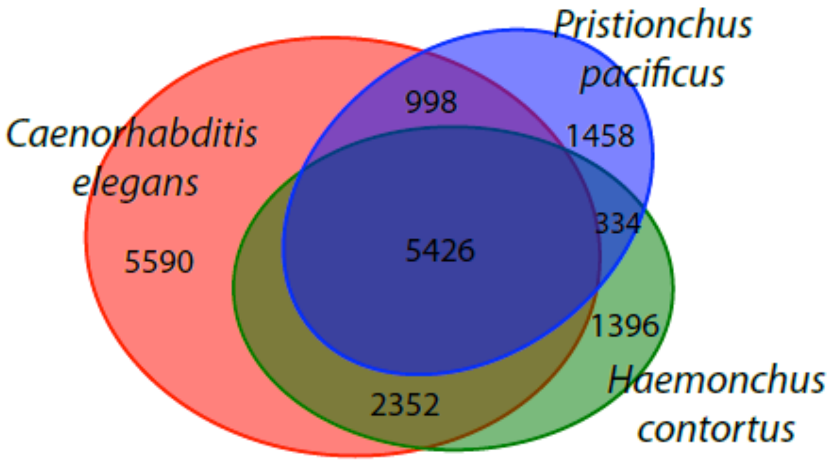

Figure S2. The pattern of gene family sharing between three clade V nematode groups, based on OrthoMCL clusters follows the expected phylogenetic pattern, showing greater similarity between *H. contortus* and *C. elegans* than either has with *P. pacificus*. Note a relatively small set of otherwise conserved gene families are apparently missing in *C. elegans*

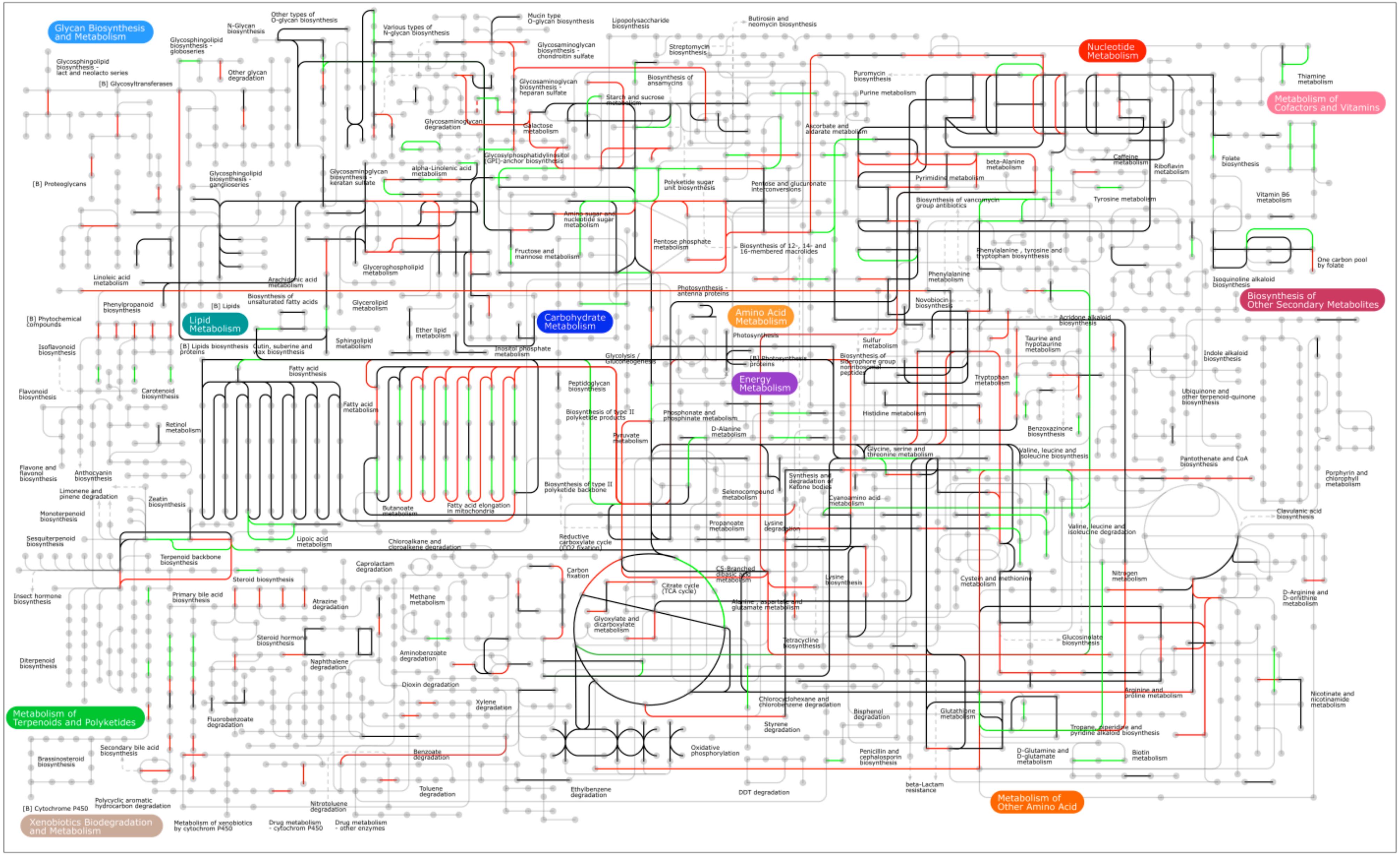

Figure S3A. Variation in metabolism across the *H. contortus* lifecycle. Metabolic networks are shown for egg to L1 transition, with significantly up-regulated genes in red, down-regulated genes in green, and genes showing no change in black. Genes not involved in *Haemonchus* metabolism are shown in grey.

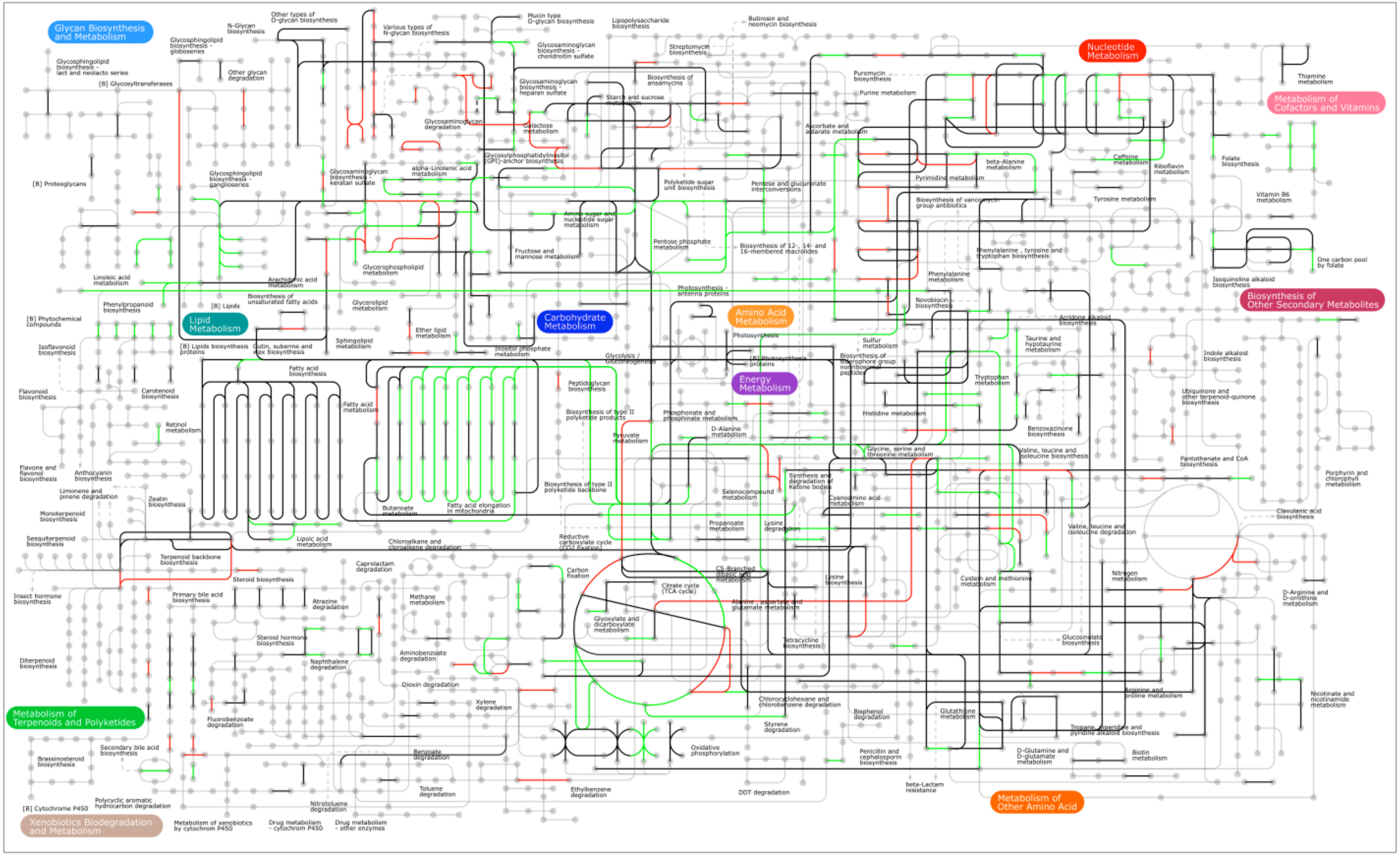

Figure S3B. Variation in metabolism across the *H. contortus* lifecycle. Metabolic networks are shown for L1 to L3 transition, with significantly up-regulated genes in red, down-regulated genes in green, and genes showing no change in black. Genes not involved in *Haemonchus* metabolism are shown in grey.

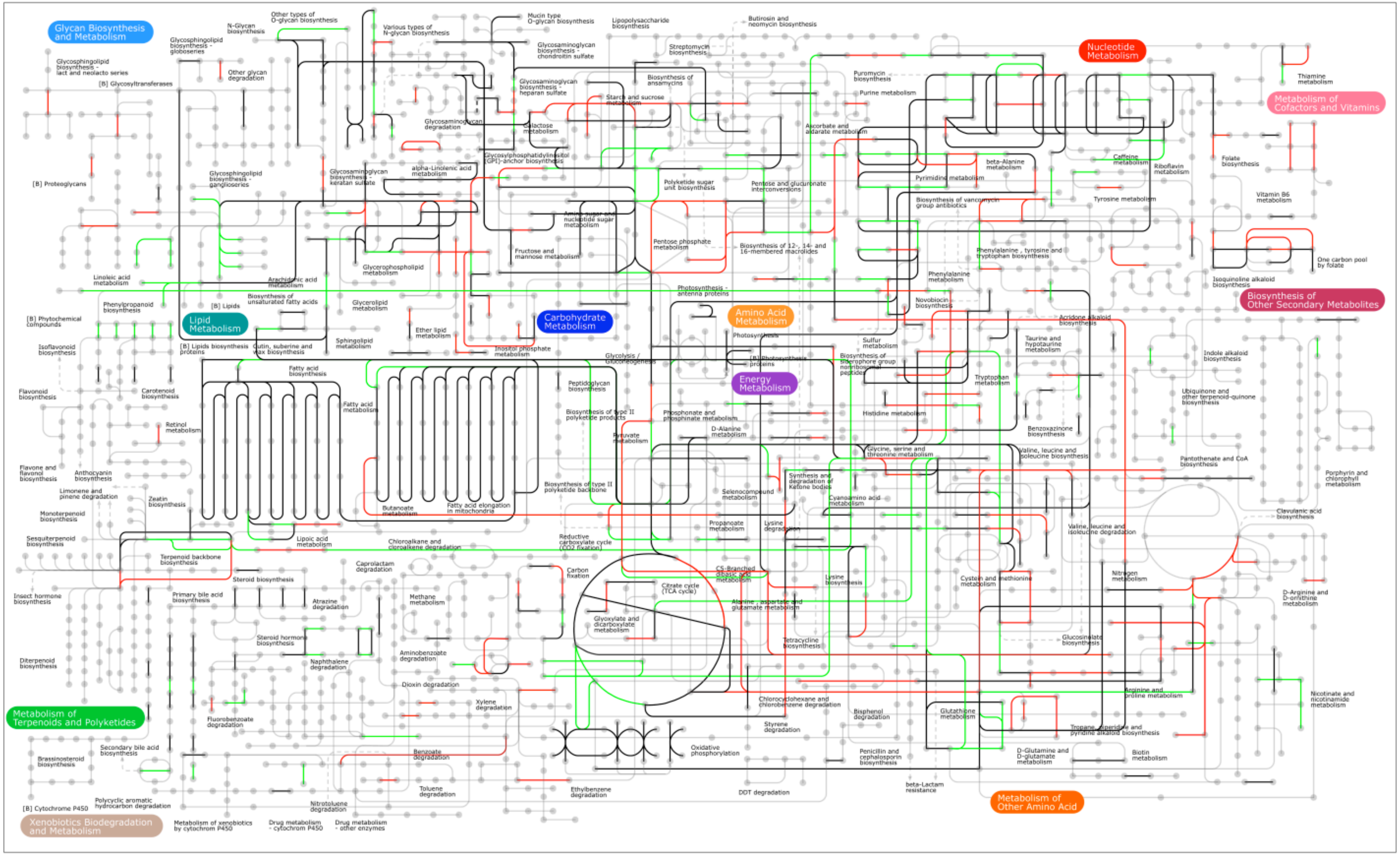

Figure S3C. Variation in metabolism across the *H. contortus* lifecycle. Metabolic networks are shown for L3 to L4 transition, with significantly up-regulated genes in red, down-regulated genes in green, and genes showing no change in black. Genes not involved in *Haemonchus* metabolism are shown in grey.

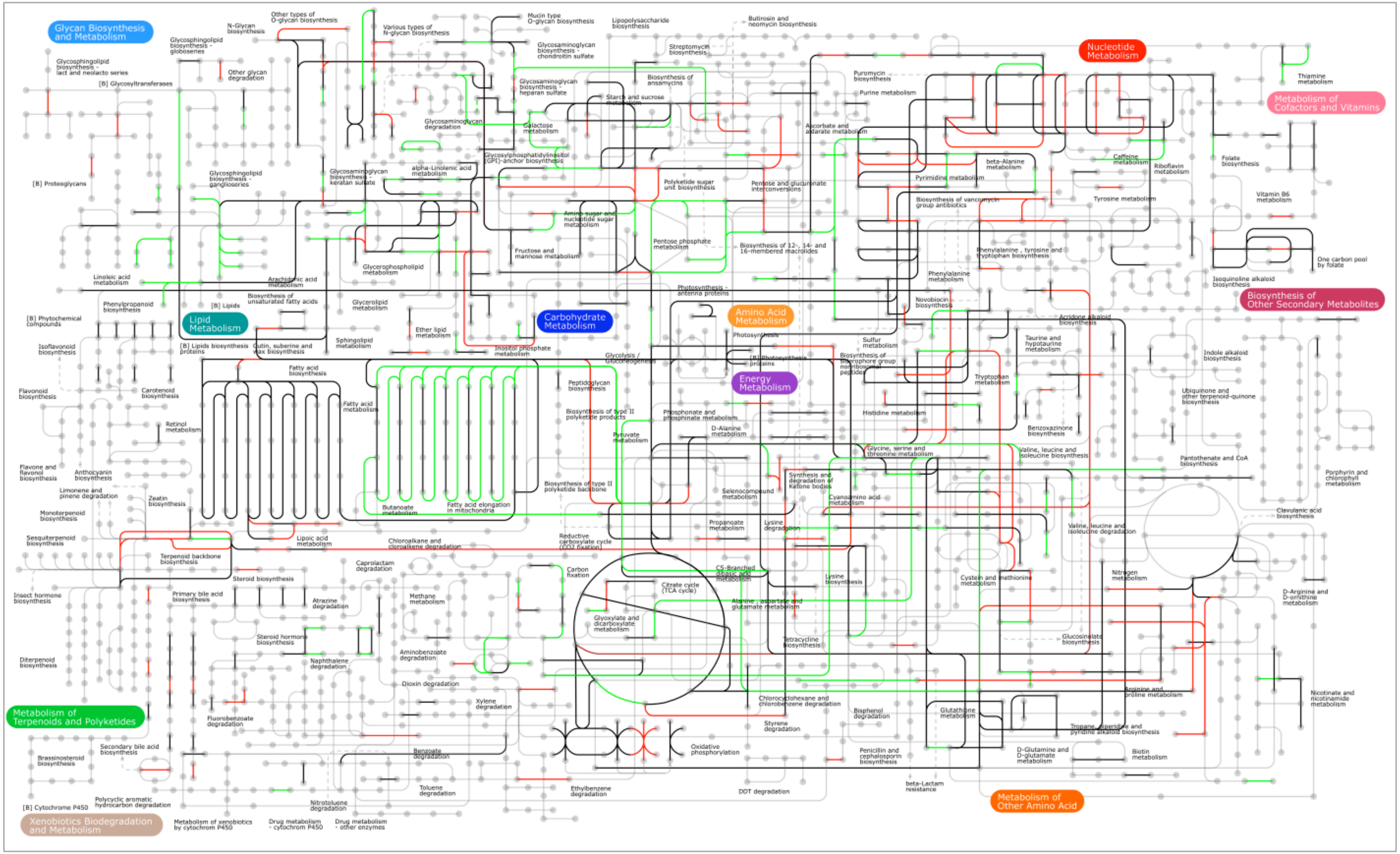

Figure S3D. Variation in metabolism across the *H. contortus* lifecycle. Metabolic networks are shown for L4 to male transition, with significantly up-regulated genes in red, down-regulated genes in green, and genes showing no change in black. Genes not involved in *Haemonchus* metabolism are shown in grey.

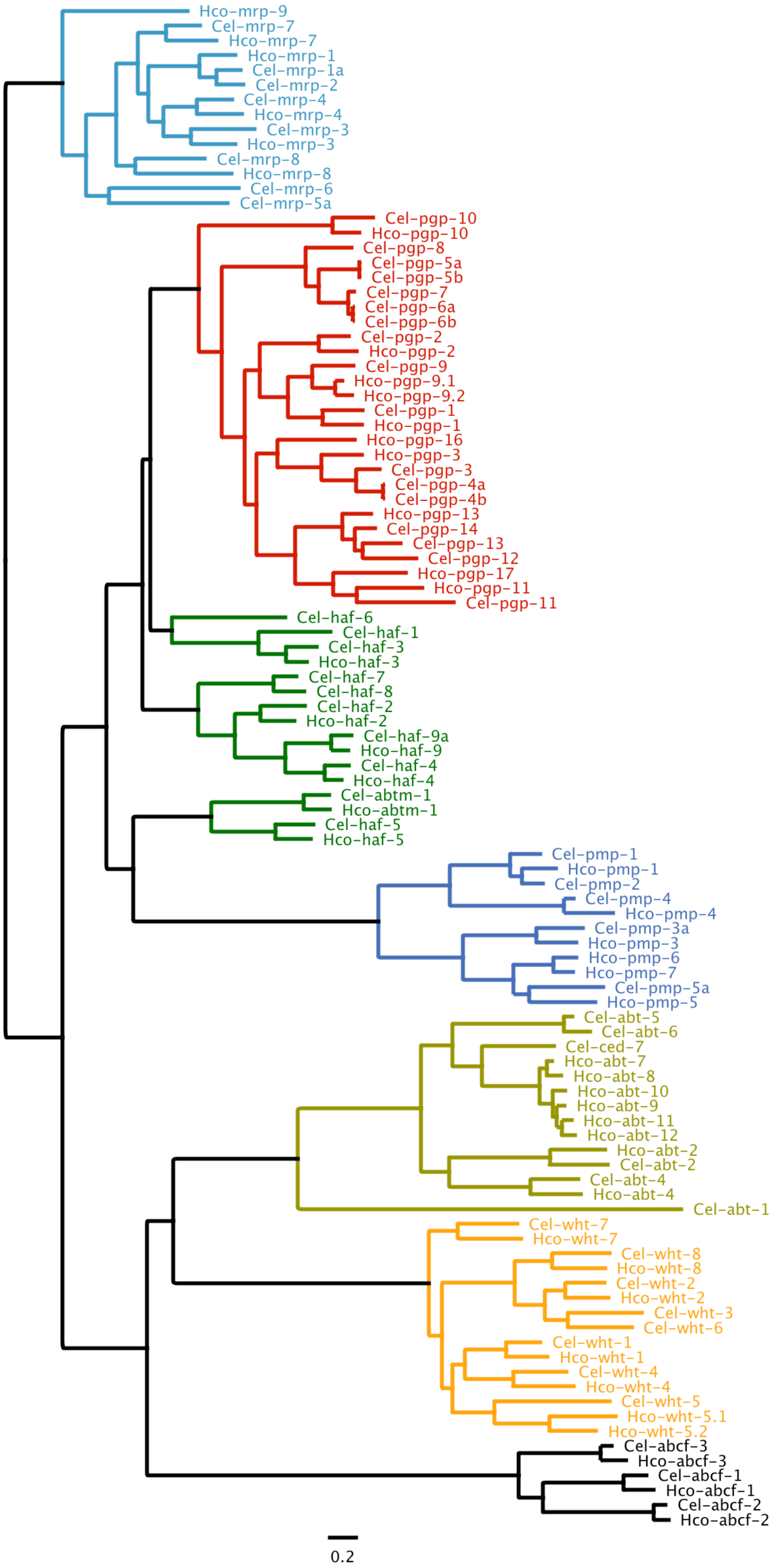

Figure S4. Maximum-likelihood phylogeny *of H. contortus* and *C. elegans* ABC transporters. Figure shows an unrooted tree for ABC transporter genes from the two nematode species, with different colours representing different transporter families: mrp (blue), pgp (red), haf (green), pmp (dark blue), abt (olive) wht (orange) abcf (black).

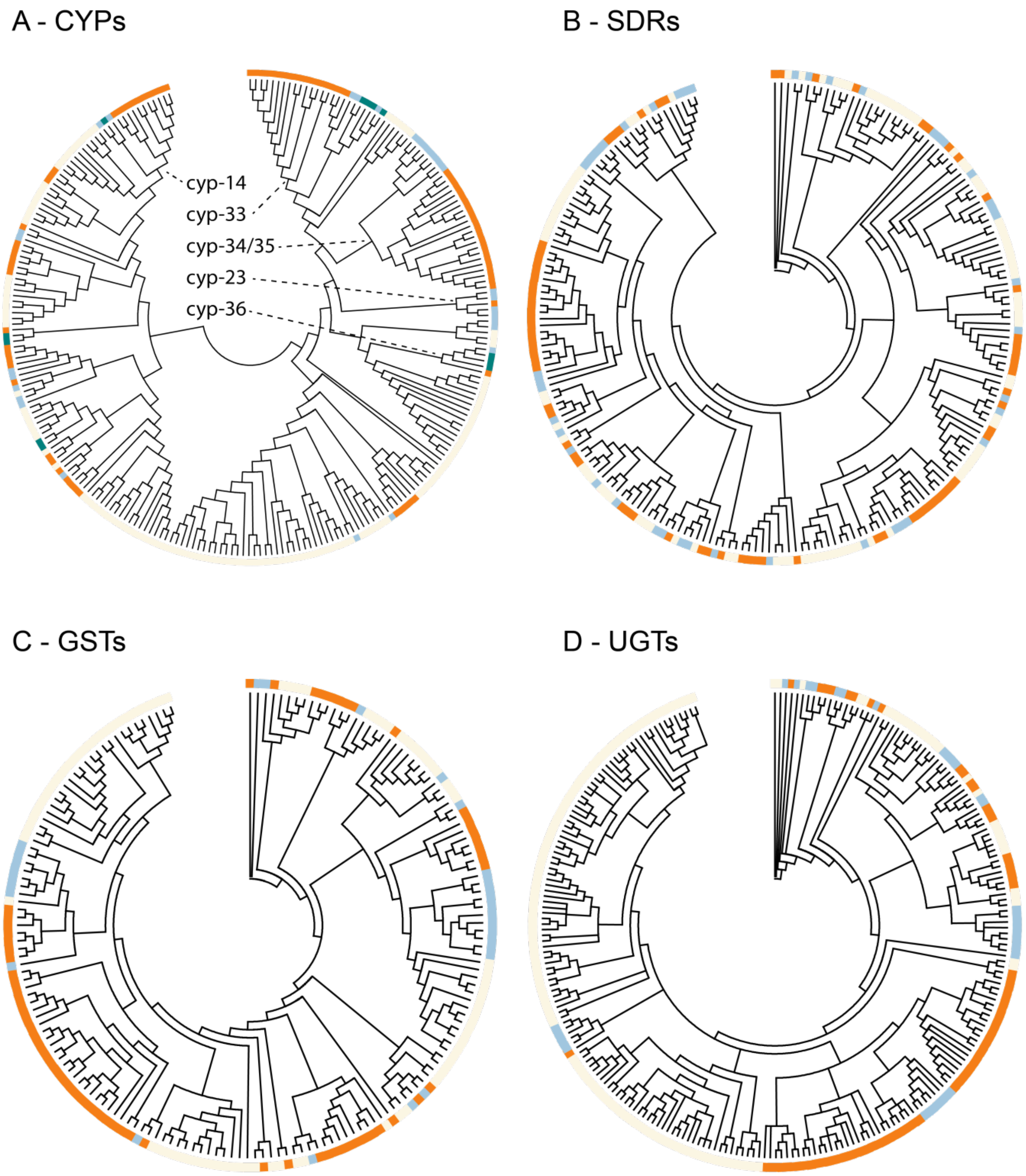

Figure S5. Phylogenetic arrangement of drug metabolism genes in *H. contortus* and related nematodes. *H. contortus* draft assembly (blue), *H. contortus* 2008 supercontigs (green), *C. elegans* (orange) and *P. pacificus* (light yellow).

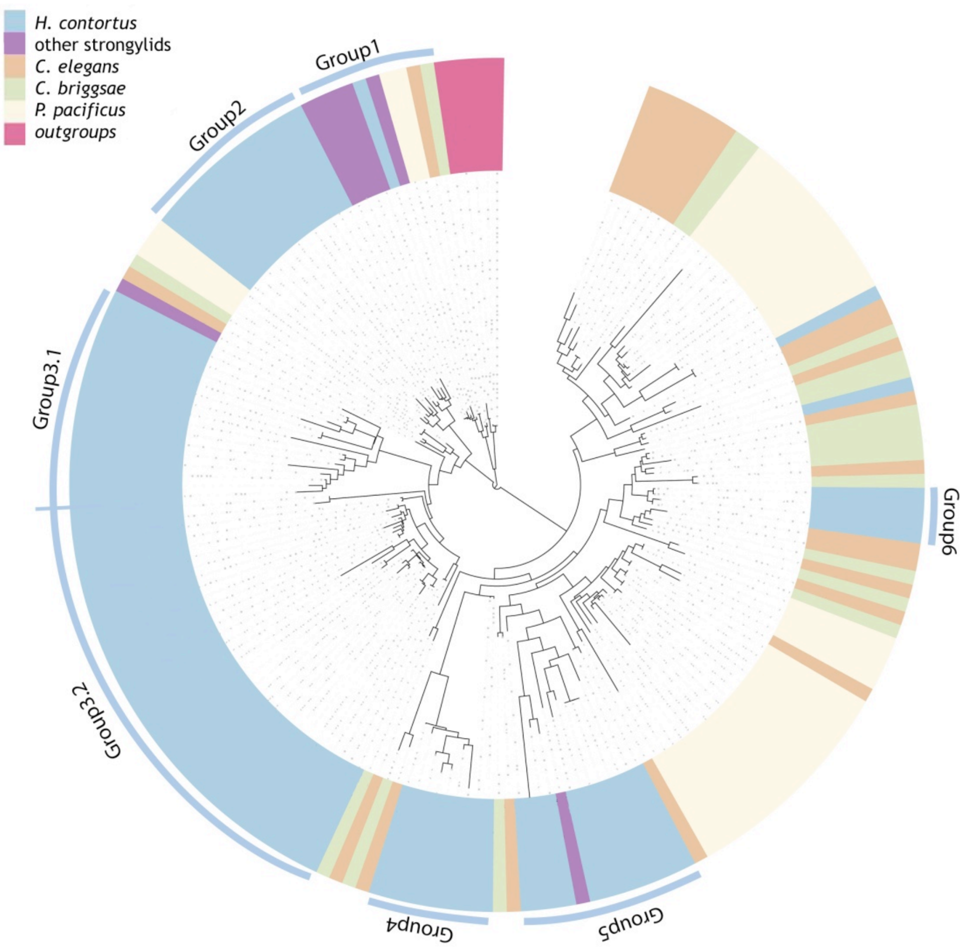

Figure S6. Phylogeny of cathepsin D aspartic protease (CTSD) genes in *H. contortus* and related nematodes. Significant expansion of CTSD is apparent in *H. contortus.* Most of the *H. contortus* sequences separate into six distinct clusters and the sequences from *P. pacificus* organise into two clusters.

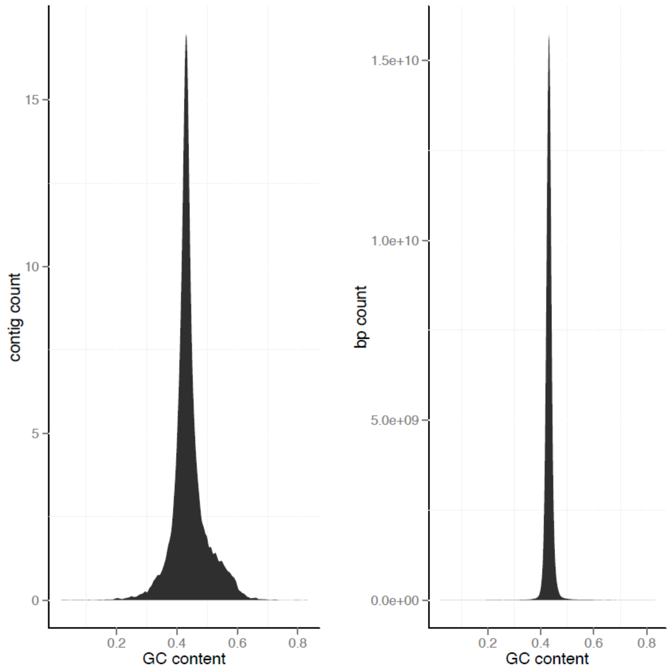

Figure S7. Base composition distribution of the *H. contortus* draft assembly, showing normal kernel density plots of the distribution of GC content between contigs and weighted by contig length, i.e. the full genomic distribution of GC content.

Table S1. Genome and gene model statistics for *H. contortus* compared to those for other published nematode genomes. Values for *M. hapla* are from [14] and those for *B. xylophilus* from [61]. Other statistics are derived from data available in Wormbase (release 221 for *M. incognita, C. elegans, P. pacificus* and *B. malayi*; release 235 for *A. suum* and *T. spiralis*). Completeness values are based on CEGs analysed with the CEGMA software package.

		Clade V			Clade IV			Clade III		Clade I
		Haemonchus contortus	*Caenorhabditis elegans*	*Pristionchus pacficus*	*Bursaphelenchus xylophilus*	*Meloidogyne hapla*	*Meloidogyne incognita*	*Brugia malayi*	*Ascaris suum*	*Trichinella spiralis*
Genome statistics	estimated genome size (Mb)	60	100	Not available	63-75	54	47-51	90-95	250	71
	haploid chromosome #	6	6	6	6	16	Varies	6	12	3
	Assembly length (Mb)	370	100	172.5	74.6	53	86	95.8	272.8	64.3
	# scaffolds	26,044	6	18,083	1,231	1,523	2,817	8,180	1,618	8,794
	scaffold N50 (kb)	83	17,493	1,244	1,158	84	83	94	408	1,739
	Longest scaffold (kb)	947	20,924	5,268	3,612	360	593	6,534	3,795	9,739
	GC content	41.3	35.4	42	40.4	27.4	31.4	30.5	37.9	34
Gene model statistics	Number of gene Models	21,799	20,056	23,500	18,074	14,420	19,212	18,348	18,542	15,808
	Gene density (genes / Mb)	59	200	136	242	272	223	192	68	246
	Mean protein length (aa)	410	440	332	345	310	354	312	327	317
	Mean/Median exon len. (bp)	126 / 105	202 / 145	97 / 85	289 / 183	172/145	169/136	160 / 138	153/137	128 / 129
	Mean/median exons/gene	9.8 / 7	6.5 / 5	10.3 /8	4.5 /4	6.1 / 4	6.6 / 5	5.9 / 3	6.4/5.0	5.78 / 4
	Mean/median intron len. (bp)	601 / 172	320/66	309/141	153 / 69	154/55	230 /82	280 / 215	1023/690	198 / 83
Completeness	CEGMA completeness (% complete/partial)	91/93	100/100	95/98	97/98	95/96	73/77	95/96	94/96	95/95
	CEG gene count (complete/partial)	1.42/1.45	1.05/1.06	1.20/1.23	1.08/1.09	1.07/1.12	1.53/1.61	1.07/1.11	1.13/1.14	1.13/1.16

Table S2. Analysis of assembly completeness, based on CEGMA v2.4.

	# Prots	% Completeness	#Total	Average	% Ortho
Complete	225	90.73	320	1.42	36.00
Group 1	58	87.88	77	1.33	27.59
Group 2	49	87.50	66	1.35	32.65
Group 3	55	90.16	86	1.56	47.27
Group 4	63	96.92	91	1.44	36.51
Partial	231	93.15	334	1.45	37.23
Group 1	61	92.42	84	1.38	29.51
Group 2	51	91.07	69	1.35	33.33
Group 3	56	91.80	89	1.59	48.21
Group 4	63	96.92	92	1.46	38.10

Table S3. RNA-seq libraries. All transcriptome libraries had fragment size of 300-400 bp and paired end reads of 100 bp.

***H. contortus* life-stage**	**isolate**	**number of reads**	**ENA accession number**
eggs	MHco3(ISE).N1	11,176,696	ERS209876
eggs	MHco3(ISE).N1	13,251,399	ERS209877
eggs	MHco3(ISE).N1	16,670,770	ERS209878
L1	MHco3(ISE).N1	13,117,727	ERS209879
L1	MHco3(ISE).N1	11,670,618	ERS209880
L1	MHco3(ISE).N1	15,327,087	ERS209881
sheathed L3	MHco3(ISE).N1	14,929,822	ERS092633
sheathed L3	MHco3(ISE).N1	14,369,393	ERS092634
sheathed L3	MHco3(ISE).N1	16,187,935	ERS092635
L4	MHco3(ISE).N1	12,074,945	ERS209882
L4	MHco3(ISE).N1	15,890,211	ERS209883
L4	MHco3(ISE).N1	14,195,344	ERS209884
adult female	MHco3(ISE).N1	11,626,701	ERS209885
adult female	MHco3(ISE).N1	14,034,068	ERS209886
adult female	MHco3(ISE).N1	11,989,468	ERS209875
adult male	MHco3(ISE).N1	16,292,131	ERS092624
adult male	MHco3(ISE).N1	15,372,590	ERS092625
adult male	MHco3(ISE).N1	16,391,491	ERS092626
dissected adult female gut	MHco3(ISE)	15,266,731	ERS092636
dissected adult female gut	MHco3(ISE)	14,533,415	ERS092637
dissected adult female gut	MHco3(ISE)	14,469,949	ERS092638

Table S4. 130 scaffolds with more than five one-to-one *C. elegans* and *H. contortus* orthologs

scaffold	no. genes on scaffold	no. one-to-one orthologs	I	II	III	IV	V	X	=	% dominating
pathogens_Hcontortus_scaffold_1	64	15	1	1	0	0	1	12	X	80.00%
pathogens_Hcontortus_scaffold_4	42	15	0	1	1	0	0	13	X	86.70%
pathogens_Hcontortus_scaffold_10	32	11	2	0	0	0	0	9	X	81.80%
pathogens_Hcontortus_scaffold_8	31	7	0	0	0	0	0	7	X	100.00%
pathogens_Hcontortus_scaffold_25	29	10	2	1	0	0	0	7	X	70.00%
pathogens_Hcontortus_scaffold_13	28	10	0	2	0	0	0	8	X	80.00%
pathogens_Hcontortus_scaffold_38	27	12	1	1	0	0	2	8	X	66.70%
pathogens_Hcontortus_scaffold_24	26	10	0	0	0	0	0	10	X	100.00%
pathogens_Hcontortus_scaffold_27	22	9	0	1	0	0	0	8	X	88.90%
pathogens_Hcontortus_scaffold_20	21	5	0	0	0	0	1	4	X	80.00%
pathogens_Hcontortus_scaffold_14	20	7	0	1	0	0	0	6	X	85.70%
pathogens_Hcontortus_scaffold_136	20	6	0	0	0	1	1	4	X	66.70%
pathogens_Hcontortus_scaffold_16	17	7	0	0	0	1	0	6	X	85.70%
pathogens_Hcontortus_scaffold_278	13	5	0	0	0	0	0	5	X	100.00%
pathogens_Hcontortus_scaffold_657	13	5	0	0	0	0	1	4	X	80.00%
pathogens_Hcontortus_scaffold_176	12	5	0	0	0	0	0	5	X	100.00%
pathogens_Hcontortus_scaffold_499	10	5	0	0	0	0	0	5	X	100.00%
pathogens_Hcontortus_scaffold_196	10	5	0	0	0	0	0	5	X	100.00%
pathogens_Hcontortus_scaffold_197	10	5	0	0	1	0	0	4	X	80.00%
pathogens_Hcontortus_scaffold_168	10	5	1	0	0	0	0	4	X	80.00%
pathogens_Hcontortus_scaffold_19	39	7	0	0	1	0	6	0	5	71.40%
pathogens_Hcontortus_scaffold_12	37	14	1	0	0	1	12	0	5	35.70%
pathogens_Hcontortus_scaffold_81	36	8	0	1	0	1	6	0	5	62.50%
pathogens_Hcontortus_scaffold_64	34	11	0	0	1	0	9	1	5	45.50%
pathogens_Hcontortus_scaffold_94	31	6	0	0	0	1	5	0	5	83.30%
pathogens_Hcontortus_scaffold_84	30	8	0	0	1	0	7	0	5	62.50%
pathogens_Hcontortus_scaffold_26	29	8	1	0	0	1	6	0	5	62.50%
pathogens_Hcontortus_scaffold_181	26	10	0	0	1	0	8	1	5	50.00%
pathogens_Hcontortus_scaffold_190	26	10	1	0	0	0	9	0	5	50.00%
pathogens_Hcontortus_scaffold_116	26	6	0	0	0	0	6	0	5	83.30%
pathogens_Hcontortus_scaffold_97	25	6	0	0	0	0	5	1	5	83.30%
pathogens_Hcontortus_scaffold_106	24	6	0	0	0	0	6	0	5	83.30%
pathogens_Hcontortus_scaffold_99	23	7	0	1	1	0	3	2	5	71.40%
pathogens_Hcontortus_scaffold_138	23	6	0	0	0	0	6	0	5	83.30%
pathogens_Hcontortus_scaffold_132	20	6	0	1	0	0	5	0	5	83.30%
pathogens_Hcontortus_scaffold_83	19	6	0	0	0	0	4	2	5	83.30%
pathogens_Hcontortus_scaffold_34	19	8	0	0	0	0	8	0	5	62.50%
pathogens_Hcontortus_scaffold_354	19	5	2	0	0	1	2	0	5	100.00%
pathogens_Hcontortus_scaffold_128	18	7	0	0	0	0	6	1	5	71.40%
pathogens_Hcontortus_scaffold_664	17	6	0	1	0	0	5	0	5	83.30%
pathogens_Hcontortus_scaffold_348	16	5	0	0	0	0	4	1	5	100.00%
pathogens_Hcontortus_scaffold_243	16	6	1	0	0	0	5	0	5	83.30%
pathogens_Hcontortus_scaffold_174	15	5	0	0	0	0	5	0	5	100.00%
pathogens_Hcontortus_scaffold_279	12	6	1	0	0	0	3	2	5	83.30%

pathogens_Hcontortus_scaffold_1003	11	5	0	1	0	0	4	0	5	100.00%
pathogens_Hcontortus_scaffold_825	10	5	0	0	0	0	5	0	5	100.00%
pathogens_Hcontortus_scaffold_429	8	5	0	0	0	1	4	0	5	100.00%
pathogens_Hcontortus_scaffold_11	31	9	0	0	0	7	1	1	4	77.80%
pathogens_Hcontortus_scaffold_129	29	6	0	0	0	6	0	0	4	100.00%
pathogens_Hcontortus_scaffold_223	23	9	1	2	0	5	1	0	4	55.60%
pathogens_Hcontortus_scaffold_60	22	9	0	1	0	8	0	0	4	88.90%
pathogens_Hcontortus_scaffold_177	18	5	0	0	0	5	0	0	4	100.00%
pathogens_Hcontortus_scaffold_741	10	5	0	0	1	4	0	0	4	80.00%
pathogens_Hcontortus_scaffold_17	42	5	0	1	4	0	0	0	3	80.00%
pathogens_Hcontortus_scaffold_62	27	7	0	0	7	0	0	0	3	100.00%
pathogens_Hcontortus_scaffold_186	14	6	0	1	5	0	0	0	3	83.30%
pathogens_Hcontortus_scaffold_490	13	5	1	0	3	0	0	1	3	60.00%
pathogens_Hcontortus_scaffold_570	13	5	0	0	4	1	0	0	3	80.00%
pathogens_Hcontortus_scaffold_75	39	19	0	16	2	0	0	1	2	84.20%
pathogens_Hcontortus_scaffold_73	32	15	1	10	3	1	0	0	2	66.70%
pathogens_Hcontortus_scaffold_49	29	6	0	6	0	0	0	0	2	100.00%
pathogens_Hcontortus_scaffold_74	29	5	0	5	0	0	0	0	2	100.00%
pathogens_Hcontortus_scaffold_63	28	9	0	8	0	0	0	1	2	88.90%
pathogens_Hcontortus_scaffold_36	28	8	0	8	0	0	0	0	2	100.00%
pathogens_Hcontortus_scaffold_6	27	6	0	5	0	1	0	0	2	83.30%
pathogens_Hcontortus_scaffold_44	27	11	0	10	1	0	0	0	2	90.90%
pathogens_Hcontortus_scaffold_22	26	5	0	4	0	0	1	0	2	80.00%
pathogens_Hcontortus_scaffold_32	26	5	1	4	0	0	0	0	2	80.00%
pathogens_Hcontortus_scaffold_61	25	6	0	3	1	1	0	1	2	50.00%
pathogens_Hcontortus_scaffold_214	25	9	0	9	0	0	0	0	2	100.00%
pathogens_Hcontortus_scaffold_145	24	7	2	5	0	0	0	0	2	71.40%
pathogens_Hcontortus_scaffold_28	24	5	1	4	0	0	0	0	2	80.00%
pathogens_Hcontortus_scaffold_35	23	11	0	9	0	1	0	1	2	81.80%
pathogens_Hcontortus_scaffold_52	22	10	0	8	0	0	1	1	2	80.00%
pathogens_Hcontortus_scaffold_7	20	6	0	5	1	0	0	0	2	83.30%
pathogens_Hcontortus_scaffold_346	20	6	0	6	0	0	0	0	2	100.00%
pathogens_Hcontortus_scaffold_388	19	6	0	5	0	1	0	0	2	83.30%
pathogens_Hcontortus_scaffold_18	18	7	0	6	0	0	0	1	2	85.70%
pathogens_Hcontortus_scaffold_95	18	6	0	4	0	2	0	0	2	66.70%
pathogens_Hcontortus_scaffold_143	18	8	0	7	0	1	0	0	2	87.50%
pathogens_Hcontortus_scaffold_47	17	8	0	6	1	0	0	1	2	75.00%
pathogens_Hcontortus_scaffold_103	17	5	1	3	0	0	1	0	2	60.00%
pathogens_Hcontortus_scaffold_504	17	7	0	7	0	0	0	0	2	100.00%
pathogens_Hcontortus_scaffold_100	17	5	0	5	0	0	0	0	2	100.00%
pathogens_Hcontortus_scaffold_120	16	7	0	5	0	1	0	1	2	71.40%
pathogens_Hcontortus_scaffold_91	16	7	1	5	1	0	0	0	2	71.40%
pathogens_Hcontortus_scaffold_566	14	6	0	4	0	1	1	0	2	66.70%
pathogens_Hcontortus_scaffold_328	14	6	0	5	0	0	1	0	2	83.30%
pathogens_Hcontortus_scaffold_309	14	7	1	6	0	0	0	0	2	85.70%
pathogens_Hcontortus_scaffold_135	14	6	0	6	0	0	0	0	2	100.00%
pathogens_Hcontortus_scaffold_514	12	5	1	3	0	0	0	1	2	60.00%
pathogens_Hcontortus_scaffold_412	12	6	1	4	1	0	0	0	2	66.70%
pathogens_Hcontortus_scaffold_834	11	5	0	5	0	0	0	0	2	100.00%
pathogens_Hcontortus_scaffold_1318	8	5	0	5	0	0	0	0	2	100.00%
pathogens_Hcontortus_scaffold_1053	7	5	0	3	0	0	0	2	2	60.00%

pathogens_Hcontortus_scaffold_183	7	5	0	4	1	0	0	0	2	80.00%
pathogens_Hcontortus_scaffold_3	51	13	1	0	1	1	0	1	1	76.90%
pathogens_Hcontortus_scaffold_69	27	6	5	0	1	0	0	0	1	83.30%
pathogens_Hcontortus_scaffold_141	27	8	8	0	0	0	0	0	1	100.00%
pathogens_Hcontortus_scaffold_56	26	7	7	0	0	0	0	0	1	100.00%
pathogens_Hcontortus_scaffold_164	25	8	5	0	1	0	2	0	1	62.50%
pathogens_Hcontortus_scaffold_123	23	8	6	0	1	1	0	0	1	75.00%
pathogens_Hcontortus_scaffold_149	22	10	8	1	0	0	1	0	1	80.00%
pathogens_Hcontortus_scaffold_92	21	6	6	0	0	0	0	0	1	100.00%
pathogens_Hcontortus_scaffold_102	20	6	6	0	0	0	0	0	1	100.00%
pathogens_Hcontortus_scaffold_119	18	8	5	2	0	0	0	1	1	62.50%
pathogens_Hcontortus_scaffold_344	18	7	5	2	0	0	0	0	1	71.40%
pathogens_Hcontortus_scaffold_80	17	9	7	1	0	1	0	0	1	77.80%
pathogens_Hcontortus_scaffold_454	17	5	3	1	0	1	0	0	1	60.00%
pathogens_Hcontortus_scaffold_271	17	8	8	0	0	0	0	0	1	100.00%
pathogens_Hcontortus_scaffold_51	16	7	3	0	1	1	1	1	1	42.90%
pathogens_Hcontortus_scaffold_387	16	5	3	0	0	0	1	1	1	60.00%
pathogens_Hcontortus_scaffold_78	16	6	4	2	0	0	0	0	1	66.70%
pathogens_Hcontortus_scaffold_189	16	5	4	1	0	0	0	0	1	80.00%
pathogens_Hcontortus_scaffold_263	16	5	4	1	0	0	0	0	1	80.00%
pathogens_Hcontortus_scaffold_71	16	6	6	0	0	0	0	0	1	100.00%
pathogens_Hcontortus_scaffold_312	15	5	3	0	2	0	0	0	1	60.00%
pathogens_Hcontortus_scaffold_125	15	9	9	0	0	0	0	0	1	100.00%
pathogens_Hcontortus_scaffold_508	15	6	6	0	0	0	0	0	1	100.00%
pathogens_Hcontortus_scaffold_339	15	5	5	0	0	0	0	0	1	100.00%
pathogens_Hcontortus_scaffold_67	14	6	4	0	0	0	0	2	1	66.70%
pathogens_Hcontortus_scaffold_204	14	7	5	0	0	0	1	1	1	71.40%
pathogens_Hcontortus_scaffold_395	13	5	4	0	0	1	0	0	1	80.00%
pathogens_Hcontortus_scaffold_469	13	5	4	1	0	0	0	0	1	80.00%
pathogens_Hcontortus_scaffold_267	12	5	3	2	0	0	0	0	1	60.00%
pathogens_Hcontortus_scaffold_285	12	6	6	0	0	0	0	0	1	100.00%
pathogens_Hcontortus_scaffold_1404	10	5	4	0	1	0	0	0	1	80.00%
pathogens_Hcontortus_scaffold_335	10	7	6	1	0	0	0	0	1	85.70%
pathogens_Hcontortus_scaffold_462	8	6	5	0	0	0	0	1	1	83.30%
pathogens_Hcontortus_scaffold_696	8	5	5	0	0	0	0	0	1	100.00%

Table S5. Summary of *H. contortus* and *C. elegans* gene orthology

Orthologous relationship		Frequency
H. contortus	***C. elegans***	
1	1	5012
1	Many	241
Many	1	534
Many	Many	150

Table S6. Species-specific expansions in *H. contortus* and *C. elegans* orthologs

# Hc	# Ce	Function	Ce gene
180	1	n/a	W09C3.8
33	1	n/a	F58B4.4
14	1	SCP-like domain	B0545.3
12	1	n/a	T15B7.1
18	2	Helicase domain	K08D10.5, R03D7.2
8	1	Serine protease inhibitor	C05E4.1
7	1	Zinc metalloprotease (*nas*)	T02B11.7
7	1	n/a	Y20F4.8
13	2	RNA recognition motif	R06C1.4, R09B3.2
6	1	Nuclear hormone receptor family	H27C11.1a
6	1	ABC transporter	F56F4.6
5	1	n/a	Y48G9A.11
5	1	Aldehyde reductase	C07D8.6
5	1	n/a	K03A11.6
35	7	Aminopeptidase N	M01F1.7, T07F10.1a, W04G3.6d, Y92H12BR.6 T08B1.2c, Y69A2AR.2, Y43D4.2
5	1	Aspartyl protease	K10C2.3
5	1	Splicing factor	Y111B2A.18
3	33	Major-sperm proteins	
3	22	n/a	
3	12	7 TM chemoreceptors srsx	
2	16	Core histones: H2A, H2B, H3 & H4	
2	17	Core histones: H2A, H2B, H3 & H4	
2	18	7 TM chemoreceptors srd	
1	19	Cytochrome P-450	
3	170	7 TM chemoreceptors srj & str	

Table S7. Accuracies of gene predictions by Augustus. Gene prediction accuracy was evaluated at the nucleotide, exon and transcript level using 52 manually curated gene models as references.

	Nucleotide		Exon		Transcript	
	Sensitivity (%)	Specificity (%)	Sensitivity (%)	Specificity (%)	Sensitivity (%)	Specificity (%)
Augustus with *C. elegans* parameters	92.9	97.2	74.3	74.5	21.0	15.4
Augustus trained	95.9	99.2	88.8	91.7	39.0	39.0
Augustus trained + hints	95.9	99.3	95.0	94.3	57.0	55.9

Table S8. TopGO analysis of *H. contortus* genes without clear orthology to other nematode genes. Significant GO terms indicate functional categories over-represented in members of gene families found only in *H. contortus*, or *H. contortus* genes not clustered in gene families in OrthoMCL analysis when compared to the complete *H. contortus* gene set.

Biological Process terms					
GO.ID	Term	Annotated	Significant	Expected	topGO p-value
GO:0006508	proteolysis	681	231	103.5	< 1e-30
GO:0008152	metabolic process	4739	844	720.28	1.20E-07
GO:0006548	histidine catabolic process	23	12	3.5	3.80E-05
GO:0006836	neurotransmitter transport	35	15	5.32	7.80E-05
GO:0009190	cyclic nucleotide biosynthetic process	43	17	6.54	8.50E-05
GO:0006814	sodium ion transport	49	18	7.45	0.00017
GO:0006835	dicarboxylic acid transport	16	8	2.43	0.00114
GO:0005975	carbohydrate metabolic process	309	61	46.96	0.00181
GO:0015074	DNA integration	89	24	13.53	0.00283
Cellular Component terms					
GO.ID	Term	Annotated	Significant	Expected	topGO p-value
GO:0016020	membrane	2368	300	302.82	3.20E-21
GO:0005576	extracellular region	284	56	36.32	1.40E-06
GO:0005887	integral to plasma membrane	54	14	6.91	0.0065
GO:0016533	cyclin-dependent protein kinase 5 holoenzyme complex activity	4	3	0.51	0.0075
GO:0000786	nucleosome	30	9	3.84	0.0102
GO:0000785	chromatin	44	14	5.63	0.0241
GO:0030131	clathrin adaptor complex	14	5	1.79	0.025
GO:0000775	chromosome, centromeric region	6	3	0.77	0.0309
GO:0030904	retromer complex	3	2	0.38	0.0448
Molecular Function terms					
GO.ID	Term	Annotated	Significant	Expected	topGO p-value
GO:0042302	structural constituent of cuticle	183	128	33.73	< 1e-30
GO:0004190	aspartic-type endopeptidase activity	170	95	31.33	3.10E-28
GO:0004222	metalloendopeptidase activity	161	64	29.67	1.60E-10
GO:0008237	metallopeptidase activity	249	98	45.89	8.20E-08
GO:0004500	dopamine beta-monooxygenase activity	19	11	3.5	0.00014
GO:0004415	hyalurononglucosaminidase activity	8	6	1.47	0.00077
GO:0016849	phosphorus-oxygen lyase activity	43	17	7.93	0.00085
GO:0005328	neurotransmitter: sodium symporter activity	33	14	6.08	0.0012
GO:0008026	ATP-dependent helicase activity	59	19	10.87	0.00133

Table S9. *H. contortus*-specific enzymes

Enzyme Classification	Enzyme	Pathway
1.1.1.162	erythrulose reductase	
1.1.1.163	cyclopentanol dehydrogenase	
1.1.1.295	momilactone-A synthase	Diterpenoid biosynthesis
1.1.1.65	pyridoxine 4-dehydrogenase	Vitamin B6 metabolism
1.1.1.90	aryl-alcohol dehydrogenase	Tyrosine metabolism, phenylalanine metabolism, xylene degradation, toluene degradation, caprolactam degradation
1.1.4.1	vitamin-K-epoxide reductase	Ubiquinone and other terpenoid-quinone biosynthesis
1.3.3.4	protoporphyrinogen oxidase	Heme biosynthesis
1.5.3.17	non-specific polyamine oxidase;	Beta-alanine metabolism
1.6.5.4	monodehydroascorbate reductase	Ascorbate and aldarate metabolism
2.1.1.37	DNA methyltransferase	Cysteine and methionine metabolism
2.4.1.145	alpha-1,3-mannosyl-glycoprotein 4-beta-N-acetylglucosaminyltransferase	N-Glycan biosynthesis
2.4.1.174	glucuronylgalactosylproteoglycan 4-beta-N-acetylgalactosaminyltransferase	Glycosaminoglycan biosynthesis - chondroitin sulfate / dermatan sulfate
2.5.1.29	geranylgeranyl diphosphate synthase	Terpenoid backbone biosynthesis
2.6.1.18	beta-alanine-pyruvate transaminase	Valine, leucine and isoleucine degradation, beta-alanine metabolism, propanoate metabolism
2.6.1.36	L-lysine 6-transaminase	
2.7.7.39	glycerol-3-phosphate cytidylyltransferase	Glycerophospholipid metabolism
3.1.6.6	choline-sulfatase	
3.4.22.67	zingipain	
3.4.23.36	signal peptidase II	
3.4.23.45	memapsin 1	
3.4.23.46	memapsin 2	Tyrosine metabolism
3.5.1.18	succinyl-diaminopimelate desuccinylase	Lysine biosynthesis

Table S10. Comparison of enzyme expression in different life-stages

Life-stages	Number of differentially expressed enzymes	
	Up-regulated	Down-regulated
Eggs vs. L1	232	142
L1 vs. L3	104	215
L3 vs. L4	234	171
L4 vs. male	187	138
L4 vs. female	117	267
Male vs. female	82	309
Female vs. eggs	181	129
Gut vs. female	154	203

Table S11. Potential drug targets in *H. contortus* based on chokepoint analysis

Enzyme Classification	Enzyme	Pathway	Target gene	Organism
1.1.1.103	L-threonine 3-dehydrogenase	Glycine, serine and threonine metabolism		
1.14.13.82	vanillate monooxygenase	Aminobenzoate degradation		
2.4.1.15	alpha,alpha-trehalose-phosphate synthase	Starch and sucrose metabolism		
3.1.3.12	trehalose-phosphatase	Starch and sucrose metabolism	*tpp*	*B. malayi*
5.1.3.13	dTDP-4-dehydrorhamnose 3,5-epimerase	Streptomycin biosynthesis, polyketide sugar unit biosynthesis	*rmlC*	*Mycobacterium tuberculosis*

Table S12. *H. contortus* cys-loop ligand-gated ion channel genes that may be involved in anthelmintic action and resistance

Drug Class	Ion channel family	Gene (*Hco-*)	Comments
Imidazothiazole/ Tetrahydropyrimidines	Nicotinic Acetylcholine Receptors	*acr-8*	Alternative splicing has been associated with levamisole resistance [62]
		lev-1	The *H. contortus* sequence lacks a clear N-terminal signal peptide
		unc-29	There are four copies of this gene in *H. contortus*
		unc-38	
		unc-63	
Macrocylic Lactones	Glutamate-gated chloride channels	*avr-14*	
		avr-15	
		glc-2	
		glc-3	
		glc-4	
		glc-5	Not present in *C. elegans*
		glc-6	Not present in *C. elegans*; present in other trichostrongyle species.
Amino-acetonitrile derivatives [63]	Nicotinic Acetylcholine Receptors	*deg-3*	
		des-2	
		mptl-1	Most similar to the *C. elegans acr-20.* No clear ortholog of *acr-23* is present.
Spiroindoles [64]	Nicotinic Acetylcholine Receptors		Not clear, may be very similar to the imidazothiazoles in terms of the genes, but unlike those compounds, the spiroindoles are antagonists.

Table S13. Illumina paired end sequence data for genome assembly. All Illumina data was generated from the same single *H. contortus* male adult.

source DNA (genomic or WGA)	parasite material	strain	mean insert size (bases)	read length (bases)	total yield (kb)	ENA sample accession number
genomic	single adult male	MHco3(ISE).N1	370	100	35980037	ERS086777
WGA	single adult male	MHco3(ISE).N1	2906	100	24344222	ERS094850

Table S14. Shotgun and paired end 454 sequence data for genome assembly.

source DNA (genomic or WGA)	parasite material	strain	library type	mean insert size (bases)	total yield (Mb)	ENA sample accession number
genomic	adults	MHco3(ISE).N1	Shotgun	n/a	1731.2	ERS213585
genomic	adults	MHco3(ISE).N1	Shotgun	n/a	3383.4	ERS213586
genomic	adults	MHco3(ISE).N1	Paired end	3000	1000	ERS213587
genomic	adults	MHco3(ISE).N1	Paired end	8000	1023.3	ERS213588
genomic	adults	MHco3(ISE).N1	Paired end	20000	666.6	ERS213590

References

1. Huang S, Chen Z, Huang G, Yu T, Yang P, Li J, Fu Y, Yuan S, Chen S, Xu A: **HaploMerger: Reconstructing allelic relationships for polymorphic diploid genome assemblies.** *Genome Research* 2012, **22:**1581-1588.

2. Hoekstra R, Criado-Fornelio A, Fakkeldij J, Bergman J, Roos MH: **Microsatellites of the parasitic nematode *Haemonchus contortus*: polymorphism and linkage with a direct repeat.** *Molecular and Biochemical Parasitology* 1997, **89:**97-107.

3. Callaghan MJ, Beh KJ: **A middle-repetitive DNA sequence element in the sheep parasitic nematode, *Trichostrongylus colubriformis*.** *Parasitology* 1994, **109:**345-350.

4. Johnson PCD, Webster LMI, Adam A, Buckland R, Dawson DA, Keller LF: **Abundant variation in microsatellites of the parasitic nematode *Trichostrongylus tenuis* and linkage to a tandem repeat.** *Molecular and Biochemical Parasitology* 2006, **148:**210-218.

5. Grillo V, Jackson F, Gilleard JS: **Characterisation of *Teladorsagia circumcincta* microsatellites and their development as population genetic markers.** *Molecular and Biochemical Parasitology* 2006, **148:**181-189.

6. Laing R, Hunt M, Protasio AV, Saunders G, Mungall K, Laing S, Jackson F, Quail M, Beech R, Berriman M et al.: **Annotation of two large contiguous regions from the *Haemonchus contortus* genome using RNA-seq and comparative analysis with *Caenorhabditis elegans*.** *PLoS One* 2011, **6:**e23216.

7. Guiliano DB, Hall N, Jones SJ, Clark LN, Corton CH, Barrell BG, Blaxter ML: **Conservation of long-range synteny and microsynteny between the genomes of two distantly related nematodes.** *Genome Biol* 2002, **3:**RESEARCH0057.

8. Denker JA, Zuckerman DM, Maroney PA, Nilsen TW: **New components of the spliced leader RNP required for nematode trans-splicing.** *Nature* 2002, **417:**667-670.

9. Jones A, Sattelle D: **The cys-loop ligand-gated ion channel gene superfamily of the nematode, *Caenorhabditis elegans*.** *Invert Neurosci* 2008, **8:**41-47.

10. Beg AA, Jorgensen EM: **EXP-1 is an excitatory GABA-gated cation channel.** *Nat Neurosci* 2003, **6:**1145-1152.

11. Bennett HM, Williamson SM, Walsh TK, Woods DJ, Wolstenholme AJ: **ACR-26: A novel nicotinic receptor subunit of parasitic nematodes.** *Molecular and Biochemical Parasitology* 2012, **183:**151-157.

12. Jones A, Davis P, Hodgkin J, Sattelle D: **The nicotinic acetylcholine receptor gene family of the nematode *Caenorhabditis elegans*: an update on nomenclature.** *Invert Neurosci* 2007, **7:**129-131.

13. Opperman CH, Bird DM, Williamson VM, Rokhsar DS, Burke M, Cohn J, Cromer J, Diener S, Gajan J, Graham S et al.: **Sequence and genetic map of *Meloidogyne hapla*: A compact nematode genome for plant parasitism.** *Proc Natl Acad Sci U S A* 2008, **105:**14802-14807.

14. Abad P, Gouzy J, Aury JM, Castagnone-Sereno P, Danchin EG, Deleury E, Perfus-Barbeoch L, Anthouard V, Artiguenave F, Blok VC et al.: **Genome sequence of the metazoan plant-parasitic nematode *Meloidogyne incognita*.** *Nat Biotechnol* 2008, **26:**909-915.

15. Scott AL, Ghedin E: **The genome of *Brugia malayi* - All worms are not created equal.** *Parasitology International* 2009, **58:**6-11.

16. Blackhall WJ, Prichard RK, Beech RN: **P-glycoprotein selection in strains of *Haemonchus contortus* resistant to benzimidazoles.** *Vet Parasitol* 2008, **152:**101-107.

17. Dicker AJ, Nisbet AJ, Skuce PJ: **Gene expression changes in a P-glycoprotein (Tci-pgp-9) putatively associated with ivermectin resistance in *Teladorsagia circumcincta*.** *Int J Parasitol* 2011, **41:**935-942.

18. Maizels RM, Balic A, Gomez-Escobar N, Nair M, Taylor MD, Allen JE: **Helminth parasites - masters of regulation.** *Immunological Reviews* 2004, **201:**89-116.

19. Tcherepanova I, Bhattacharyya L, Rubin CS, Freedman JH: **Aspartic proteases from the nematode *Caenorhabditis elegans*. Structural organization and developmental and cell-specific expression of asp-1.** *J Biol Chem* 2000, **275:**26359-26369.

20. Maizels RM, Hewitson JP, Murray J, Harcus YM, Dayer B, Filbey KJ, Grainger JR, McSorley HJ, Reynolds LA, Smith KA: **Immune modulation and modulators in *Heligmosomoides polygyrus* infection.** *Experimental Parasitology* 2012, **132:**76-89.

21. Roos MH, Otsen M, Hoekstra R, Veenstra JG, Lenstra JA: **Genetic analysis of inbreeding of two strains of the parasitic nematode *Haemonchus contortus*.** *International Journal for Parasitology* 2004, **34:**109-115.

22. Jackson F, Hoste H: ***In vitro* methods for the primary screening of plant products for direct activity against ruminant gastrointestinal nematodes.** In *In vitro screening of plant resources for extra-nutritional attributes in ruminants: nuclear and related methodologies*. Edited by Vercoe PE, Makkar HPS, Schlink AC. Springer Netherlands; 2010:25-45.

23. Rehman A, Jasmer DP: **A tissue specific approach for analysis of membrane and secreted protein antigens from *Haemonchus contortus* gut and its application to diverse nematode species.** *Mol Biochem Parasitol* 1998, **97:**55-68.

24. Kozarewa I, Ning Z, Quail MA, Sanders MJ, Berriman M, Turner DJ: **Amplification-free Illumina sequencing-library preparation facilitates improved mapping and assembly of (G+C)-biased genomes.** *Nat Methods* 2009, **6:**291-295.

25. Park et al.: **An improved approach to mate-paired library preparation for Illumina sequencing** *Methods in Next Generation,* in press.

26. **Illumina** [https://icom.illumina.com/]

27. Redman E, Packard E, Grillo V, Smith J, Jackson F, Gilleard JS: **Microsatellite analysis reveals marked genetic differentiation between *Haemonchus contortus* laboratory isolates and provides a rapid system of genetic fingerprinting.** *Int J Parasitol* 2008, **38:**111-122.

28. **Roche 454** [www.454.com]

29. Myers EW, Sutton GG, Delcher AL, Dew IM, Fasulo DP, Flanigan MJ, Kravitz SA, Mobarry CM, Reinert KHJ, Remington KA et al.: **A Whole-Genome Assembly of *Drosophila*.** *Science* 2000, **287:**2196-2204.

30. Simpson JT, Durbin R: **Efficient *de novo* assembly of large genomes using compressed data structures.** *Genome Research* 2012, **22:**549-556.

31. Kurtz S, Narechania A, Stein J, Ware D: **A new method to compute K-mer frequencies and its application to annotate large repetitive plant genomes.** *BMC Genomics* 2008, **9:**517.

32. Zerbino DR, Birney E: **Velvet: Algorithms for de novo short read assembly using de Bruijn graphs.** *Genome Research* 2008, **18:**821-829.

33. Yao G, Ye L, Gao H, Minx P, Warren WC, Weinstock GM: **Graph accordance of next-generation sequence assemblies.** *Bioinformatics* 2012, **28:**13-16.

34. Boetzer M, Pirovano W: **Toward almost closed genomes with GapFiller.** *Genome Biology* 2012, **13:**R56.

35. Tsai I, Otto T, Berriman M: **Improving draft assemblies by iterative mapping and assembly of short reads to eliminate gaps.** *Genome Biology* 2010, **11:**R41.

36. Boetzer M, Henkel CV, Jansen HJ, Butler D, Pirovano W: **Scaffolding pre-assembled contigs using SSPACE.** *Bioinformatics* 2011, **27:**578-579.

37. Barriere A, Yang SP, Pekarek E, Thomas CG, Haag ES, Ruvinsky I: **Detecting heterozygosity in shotgun genome assemblies: Lessons from obligately outcrossing nematodes.** *Genome Res* 2009, **19:**470-480.

38. Ostlund G, Schmitt T, Forslund K, Kostler T, Messina DN, Roopra S, Frings O, Sonnhammer EL: **InParanoid 7: new algorithms and tools for eukaryotic orthology analysis.** *Nucleic Acids Res* 2010, **38:**D196-D203.

39. Li L, Stoeckert CJ, Roos DS: **OrthoMCL: Identification of Ortholog Groups for Eukaryotic Genomes.** *Genome Research* 2003, **13:**2178-2189.

40. Katoh K, Misawa K, Kuma K, Miyata T: **MAFFT: a novel method for rapid multiple sequence alignment based on fast Fourier transform.** *Nucleic Acids Research* 2002, **30:**3059-3066.

41. Capella-Gutierrez S, Silla-Martinez JM, Gabaldon T: **trimAl: a tool for automated alignment trimming in large-scale phylogenetic analyses.** *Bioinformatics* 2009, **25:**1972-1973.

42. Stamatakis A, Ludwig T, Meier H: **RAxML-III: a fast program for maximum likelihood-based inference of large phylogenetic trees.** *Bioinformatics* 2005, **21:**456-463.

43. Retief JD: **Phylogenetic analysis using PHYLIP.** *Methods Mol Biol* 2000, **132:**243-258.

44. Blumenthal T, Evans D, Link CD, Guffanti A, Lawson D, Thierry-Mieg J, Thierry-Mieg D, Chiu WL, Duke K, Kiraly M et al.: **A global analysis of *Caenorhabditis elegans* operons.** *Nature* 2002, **417:**851-854.

45. Moriya Y, Itoh M, Okuda S, Yoshizawa AC, Kanehisa M: **KAAS: an automatic genome annotation and pathway reconstruction server.** *Nucleic Acids Research* 2007, **35:**W182-W185.

46. Hung SS, Wasmuth J, Sanford C, Parkinson J: **DETECT – A Density Estimation Tool for Enzyme Classification and its application to *Plasmodium falciparum*.** *Bioinformatics* 2010, **26:**1690-1698.

47. Arakaki A, Huang Y, Skolnick J: **EFICAz2: enzyme function inference by a combined approach enhanced by machine learning.** *BMC Bioinformatics* 2009, **10:**107.

48. Yeh I, Hanekamp T, Tsoka S, Karp PD, Altman RB: **Computational Analysis of *Plasmodium falciparum* Metabolism: Organizing Genomic Information to Facilitate Drug Discovery.** *Genome Research* 2004, **14:**917-924.

49. Zhu F, Shi Z, Qin C, Tao L, Liu X, Xu F, Zhang L, Song Y, Liu X, Zhang J et al.: **Therapeutic target database update 2012: a resource for facilitating target-oriented drug discovery.** *Nucleic Acids Research* 2012, **40:**D1128-D1136.

50. Yamada T, Letunic I, Okuda S, Kanehisa M, Bork P: **iPath2.0: interactive pathway explorer.** *Nucleic Acids Research* 2011, **39:**W412-W415.

51. Finn RD, Clements J, Eddy SR: **HMMER web server: interactive sequence similarity searching.** *Nucleic Acids Research* 2011, **39:**W29-W37.

52. Bailey TL, Boden M, Buske FA, Frith M, Grant CE, Clementi L, Ren J, Li WW, Noble WS: **MEME Suite: tools for motif discovery and searching.** *Nucleic Acids Research* 2009, **37:**W202-W208.

53. Bailey TL, Gribskov M: **Combining evidence using p-values: application to sequence homology searches.** *Bioinformatics* 1998, **14:**48-54.

54. Guindon S, Gascuel O: **A simple, fast, and accurate algorithm to estimate large phylogenies by maximum likelihood.** *Syst Biol* 2003, **52:**696-704.

55. Whelan S, Goldman N: **A General Empirical Model of Protein Evolution Derived from Multiple Protein Families Using a Maximum-Likelihood Approach.** *Molecular Biology and Evolution* 2001, **18:**691-699.

56. Guindon S, Dufayard JF, Lefort V, Anisimova M, Hordijk W, Gascuel O: **New algorithms and methods to estimate maximum-likelihood phylogenies: assessing the performance of PhyML 3.0.** *Syst Biol* 2010, **59:**307-321.

57. **ImmPort database** [http://immport.niaid.nih.gov]

58. Lynn DJ, Winsor GL, Chan C, Richard N, Laird MR, Barsky A, Gardy JL, Roche FM, Chan THW, Shah N et al.: **InnateDB: facilitating systems-level analyses of the mammalian innate immune response.** *Mol Syst Biol* 2008, **4**.

59. Katoh K, Standley DM: **MAFFT Multiple Sequence Alignment Software Version 7: Improvements in Performance and Usability.** *Molecular Biology and Evolution* 2013.

60. Le SQ, Gascuel O: **An Improved General Amino Acid Replacement Matrix.** *Molecular Biology and Evolution* 2008, **25:**1307-1320.

61. Kikuchi T, Cotton JA, Dalzell JJ, Hasegawa K, Kanzaki N, McVeigh P, Takanashi T, Tsai IJ, Assefa SA, Cock PJ et al.: **Genomic insights into the origin of parasitism in the emerging plant pathogen *Bursaphelenchus xylophilus*.** *PLoS Pathog* 2011, **7:**e1002219.

62. Fauvin A, Charvet C, Issouf M, Cortet J, Cabaret J, Neveu C: **cDNA-AFLP analysis in levamisole-resistant *Haemonchus contortus* reveals alternative splicing in a nicotinic acetylcholine receptor subunit.** *Mol Biochem Parasitol* 2010, **170:**105-107.

63. Kaminsky R, Ducray P, Jung M, Clover R, Rufener L, Bouvier J, Weber SS, Wenger A, Wieland-Berghausen S, Goebel T et al.: **A new class of anthelmintics effective against drug-resistant nematodes.** *Nature* 2008, **452:**176-180.

64. Little PR, Hodge A, Maeder SJ, Wirtherle NC, Nicholas DR, Cox GG, Conder GA: **Efficacy of a combined oral formulation of derquantel-abamectin against the adult and larval stages of nematodes in sheep, including anthelmintic-resistant strains.** *Vet Parasitol* 2011, **181:**180-193.
